# Cholesterol Metabolism by Uncultured Human Gut Bacteria Influences Host Cholesterol Level

**DOI:** 10.1016/j.chom.2020.05.013

**Published:** 2020-08-12

**Authors:** Douglas J. Kenny, Damian R. Plichta, Dmitry Shungin, Nitzan Koppel, A. Brantley Hall, Beverly Fu, Ramachandran S. Vasan, Stanley Y. Shaw, Hera Vlamakis, Emily P. Balskus, Ramnik J. Xavier

**Affiliations:** 1 Broad Institute of MIT and Harvard, Cambridge, MA, USA; 2 Department of Chemistry and Chemical Biology, Harvard University, Cambridge, MA, USA; 3 Boston University and NHLBI's Framingham Heart Study, Framingham, MA, USA; 4 Sections of Preventive Medicine and Epidemiology and Cardiovascular Medicine, Departments of Medicine and Epidemiology, Boston University Schools of Medicine and Public Health, Boston, MA, USA; 5 Harvard Medical School, Boston, MA, USA; 6 Center for Microbiome Informatics and Therapeutics, Massachusetts Institute of Technology, Cambridge, MA, USA; 7 Center for Computational and Integrative Biology and Department of Molecular Biology, Massachusetts General Hospital and Harvard Medical School, Boston, MA, USA; 8 HMS and Division of Cardiovascular Medicine, Brigham and Women’s Hospital, Boston, MA; 9 Department of Odontology, Umeå University, Umeå, Sweden

**Keywords:** microbiome, cholesterol, coprostanol, hydroxysteroid dehydrogenase, microbial dark matter, metabolomics, metagenomics, metagenomic species, Clostridium cluster IV, Framingham Heart Study

## Abstract

The human microbiome encodes extensive metabolic capabilities, but our understanding of the mechanisms linking gut microbes to human metabolism remains limited. Here, we focus on the conversion of cholesterol to the poorly absorbed sterol coprostanol by the gut microbiota to develop a framework for the identification of functional enzymes and microbes. By integrating paired metagenomics and metabolomics data from existing cohorts with biochemical knowledge and experimentation, we predict and validate a group of microbial cholesterol dehydrogenases that contribute to coprostanol formation. These enzymes are encoded by *ismA* genes in a clade of uncultured microorganisms, which are prevalent in geographically diverse human cohorts. Individuals harboring coprostanol-forming microbes have significantly lower fecal cholesterol levels and lower serum total cholesterol with effects comparable to those attributed to variations in lipid homeostasis genes. Thus, cholesterol metabolism by these microbes may play important roles in reducing intestinal and serum cholesterol concentrations, directly impacting human health.

## Highlights

• Bioinformatics enabled discovery of *ismA*, a microbial cholesterol dehydrogenase
• Metagenomic species with *ismA* genes form coprostanol in microbial communities
• *ismA+* species are associated with decreased fecal and serum cholesterol in humans
• Effect sizes of *ismA+* species on serum cholesterol are on par with human genetics

## Graphical Abstract

**Figure:**
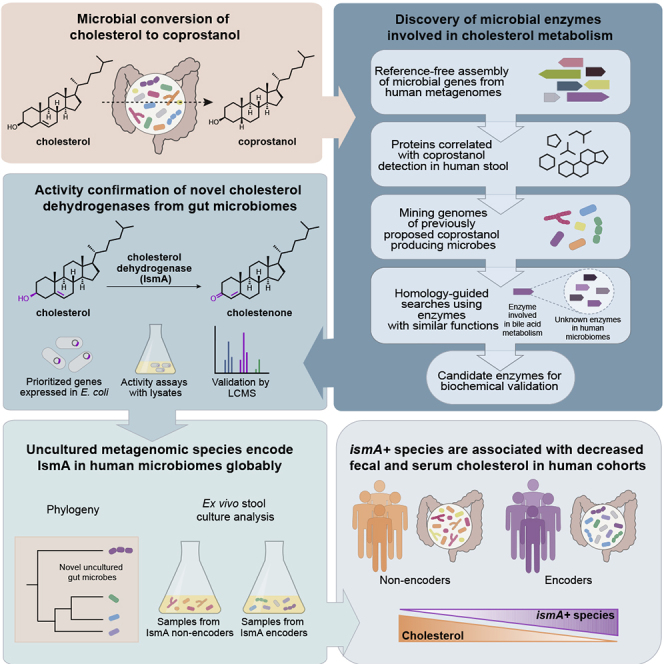

## Teaser

The consequences of cholesterol metabolism by the gut microbiome were largely unknown. Kenny, Plichta et al. identified and characterized microbial cholesterol dehydrogenase enzymes encoded by uncultured metagenomic species of bacteria. The presence of these cholesterol-metabolizing bacteria is associated with decreased stool and serum total cholesterol in human cohorts.

## Introduction

**Figure 1:**
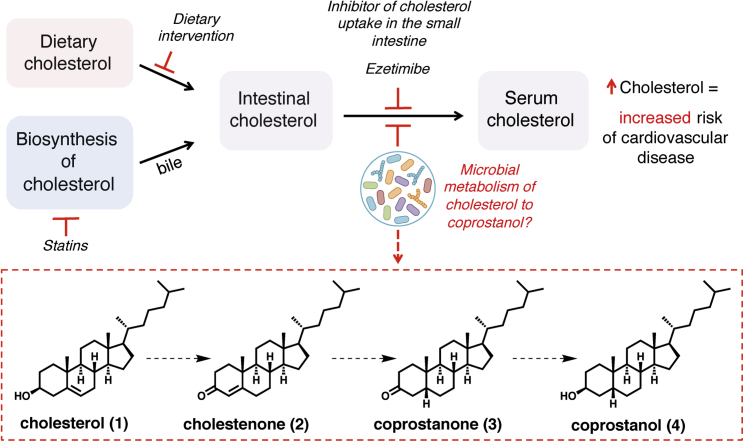
Levels of Serum Cholesterol Are Important for Human Health and Can Be Modulated by a Variety of Factors, Including the Potential Metabolism of Cholesterol by the Gut Microbiota Intestinal cholesterol levels are influenced by both dietary and host-derived cholesterol. Intervention by changes in diet or use of statins both affect levels of intestinal cholesterol, while the use of ezetimibe blocks uptake of intestinal cholesterol. Gut microbial metabolism of cholesterol may also serve to reduce cholesterol absorption in the intestine, resulting in lower serum cholesterol levels. The proposed pathway for microbial conversion of cholesterol (1) to coprostanol (4) in the microbiota involves the intermediates cholestenone (2) and coprostanone (3).

Cholesterol is a key biological molecule that functions as a structural component of all animal cell membranes and is a precursor of steroid hormones, vitamin D, and bile acids (Goldstein and Brown, 2015). Two main sources of cholesterol are thought to influence concentrations of this metabolite in serum: endogenous cholesterol synthesized in the liver and exogenous cholesterol derived from dietary components of animal origin (Figure 1) (Bays et al., 2008). The cholesterol synthesized in hepatocytes is transported to the gallbladder and is then secreted into the small intestine along with other bile salts. In the intestine, biliary cholesterol (~1–2 g/day) mixes with dietary cholesterol (~0.2–0.4 g/day in the average American diet), and both sources are eventually transported into enterocytes for packaging into lipoprotein particles and secretion into the plasma (Bays et al., 2008, Chiang, 2009, Cohen, 2008, Xu et al., 2018). Hypercholesterolemia, or high circulating cholesterol, is strongly associated with the development and progression of cardiovascular disease (CVD), which is the cause of one-fourth of all deaths in industrialized countries (Goldstein and Brown, 2015, Nordestgaard and Varbo, 2014, Rader and Hovingh, 2014). Notably, reducing cholesterol transport in the intestine is a clinically validated strategy for lowering serum cholesterol levels as demonstrated by ezetimibe, an FDA-approved small molecule inhibitor of the intestinal cholesterol transporter (Figure 1) (Bays et al., 2008).

A range of gut microbes metabolize and modify dietary and host-derived molecules in the small intestine (Koppel et al., 2017, Rowland et al., 2018). Because both sources of cholesterol pass through this environment, it has been proposed that the gut microbiota may influence serum cholesterol levels (Kriaa et al., 2019). Indeed, studies examining associations between gut microbial community composition and circulating cholesterol concentrations have shown that taking into account the abundances of particular gut bacteria can improve models that predict blood cholesterol level, and microbiota transfer from human donors with elevated serum cholesterol levels can impart this cholesterol phenotype to mice (Fu et al., 2015, Le Roy et al., 2019, Rothschild et al., 2018). Other studies have reported that administering particular bacterial species as probiotics can have cholesterol-lowering effects on the host (Park et al., 2018). However, the precise mechanisms underlying these observations are currently unknown.

A long-standing proposal for how the gut microbiota may exert cholesterol-lowering effects is through the direct metabolism of intestinal cholesterol to coprostanol (Figure 1), which would reduce the amount of cholesterol absorbed from the intestine. This microbiota-dependent reductive transformation has been known to occur in humans since the early 1900s (Flint, 1897, Rosenfeld et al., 1954, Rosenheim and Webster, 1935). The first bacterium reported to convert intestinal cholesterol to coprostanol was isolated from the cecal contents of a rat in 1973 (Eyssen et al., 1973). Based on biochemical classification at the time, this bacterium was assigned to the genus *Eubacterium*; however, numerous validly published species have been misclassified within this genus making this assignment subject to speculation (Ludwig et al., 2015). Since this initial discovery, coprostanol-generating gut bacteria with similar physical and biochemical characteristics have been reported from a variety of different sources including rats, baboons, and humans (Eyssen et al., 1973, Mott and Brinkley, 1979, Sadzikowski et al., 1977). However, most of these strains are not currently available and were never sequenced. Early work showed that coprostanol formation by this group of gut bacteria proceeds through an indirect reduction pathway involving the initial oxidation of cholesterol (1) to cholestenone (2), followed by reduction of the Δ^4,5^ double bond to form coprostanone (3), and subsequent re-reduction of the ketone to generate coprostanol (4) (Figure 1). The bacterial enzymes responsible for this metabolism were never identified (Björkhem and Gustafsson, 1971, Eyssen et al., 1973, Ren et al., 1996). More recently, other reports have implicated additional, phylogenetically diverse gut bacteria in coprostanol formation, confounding our understanding of which organisms are responsible for this metabolism in humans (Gérard et al., 2007, Lye et al., 2010).

While efforts to elucidate how gut microbial metabolism of cholesterol affects human serum cholesterol levels span over 100 years, mechanistic support for this connection has remained elusive due to a limited understanding of the gut microbes, genes, and enzymes responsible for coprostanol formation. Here, we describe a multi-disciplinary strategy for enzyme discovery used to identify and characterize a widespread family of cholesterol dehydrogenase enzymes from a clade of uncultured gut bacteria that mediate the metabolism of cholesterol to coprostanol in the gastrointestinal tract. We find that the presence of these intestinal sterol metabolism A genes (*ismA*) in a microbiome is associated with the presence of coprostanol in stool and reduced stool cholesterol levels. Finally, to demonstrate the potential for these cholesterol-metabolizing bacteria to influence human health, we show that the presence of *ismA* genes in human metagenomes is significantly associated with a decrease in total cholesterol concentrations in serum that is on par with the effects observed from variants in human genes involved in lipid homeostasis. Together, our findings support a role for gut bacterial metabolism in modulating host cholesterol levels and lay the groundwork for microbiota-targeted interventions.

## Results

### Identification of Putative Cholesterol-Metabolizing Enzymes in Human Gut Microbiome Assemblies

**Figure 2:**
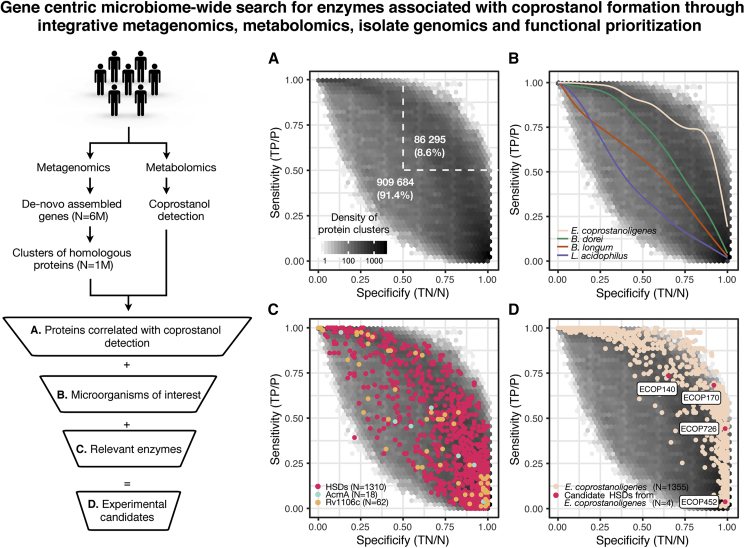
Integrative Analysis of Metagenomes, Metabolomes, Isolate Genomes, and Enzymatic Functions Reveals Candidate Bacterial Genes Involved in Cholesterol Metabolism in the Human Gut Microbiome Human gut microbiome genes from a *de novo* assembled gene catalog, after additional clustering step into groups of homologous proteins (at least 50% aa identity), were correlated with coprostanol detection in paired metagenomic-metabolomic samples and further prioritized by incorporating information from relevant microorganisms and enzymes. (A) Scores of specificity and sensitivity in relation to presence of coprostanol were calculated for each cluster of homologous proteins, and their density is represented through hexagonal bin plot; 8.6% of protein clusters are found with greater than 50% specificity and sensitivity to coprostanol detection. (B) Proteins encoded by gut microbes of interest (implicated in coprostanol formation in the literature) were used to query the clusters of homologous proteins. Clusters containing proteins with >50% aa identity to proteins found within a specified organism were used to generate a smoothed trend line (see Figure S1E for the location of species matched clusters). According to the location of these trend lines, *E. coprostanoligenes* matching clusters are more specifically associated with coprostanol formation than clusters from other microbes. (C) Clusters of homologous proteins were queried with characterized enzymes known to either catalyze the oxidation of cholesterol to cholestenone: cholesterol oxidases (PF09129), AcmA from *S. denitrificans*, and Rv1106c from *M. tuberculosis* or enzymes that can perform very similar chemical transformations (HSDs: RUMGNA_00694, Elen_1325, Elen_0198, and KGH18088). USEARCH ublast (Edgar, 2010) analysis was performed with inclusive cutoffs (>25% aa identity and 50% coverage). (D) Combining the evidence from (A)–(C), 4 putative HSDs in *E. coprostanoligenes* were identified, 3 of which (ECOP170, ECOP726, and ECOP442) had high specificity with regard to the presence of coprostanol (>0.9), albeit with greatly varying sensitivity. All four enzymes were chosen for further biochemical validation. All panels based on dataset 1 analysis; see Figures S1A–S1D for dataset 2 analysis. See also Figure S1 and Table S1.

We set out to discover the putative gut organisms and enzymes responsible for converting cholesterol to coprostanol in the human gut microbiota. To do so, we used a three-tiered, multi-disciplinary analysis consisting of (1) integration of large-scale human stool microbiome and metabolomics datasets, (2) mining genomes of previously proposed coprostanol-producing microbes, and (3) employing biochemical knowledge to prioritize enzymes with catalytic capabilities needed to metabolize cholesterol (Figure 2).

We began by analyzing gut microbiomes for enzyme-encoding genes associated with the presence of coprostanol in stool metabolomes. To avoid constraints imposed by reference genomes, which do not represent the full spectrum of microbiome enzymatic diversity, we performed *de novo* assembly of gut microbiome datasets from geographically diverse locations (n = 3,097) resulting in 5,929,528 non-redundant complete genes. These were grouped into clusters of homologous proteins (sequence-based homology, minimum 50% aa identity) in order to connect proteins with similar molecular functions and facilitate integrative analysis with stool metabolomics readouts (Suzek et al., 2015). The 50% aa identity cutoff mirrors the lowest identity threshold in the well-established UniProt framework; it was shown to efficiently group proteins with similar molecular function, but divergent sequences, together (Suzek et al., 2015). A total of 625 samples from two independent datasets had paired fecal metagenomics and metabolomics measurements (Franzosa et al., 2019, Lloyd-Price et al., 2019). To find proteins associated with coprostanol production *in vivo*, we correlated the presence of homologous protein clusters to the presence of coprostanol across these samples (Figures 2A and S1A) and derived metrics of specificity and sensitivity that represent how well the presence or absence of a protein cluster corresponds to the detection of coprostanol. In dataset 1, 91% of tested protein clusters (~909 k) had specificity or sensitivity <0.5, showing poor correlation with coprostanol; similarly, no obvious candidate cluster with both specificity and sensitivity close to 1 was revealed (Figure 2A). Overall, a similar distribution of coprostanol to protein clusters was observed in dataset 2 (Figure S1A). One potential explanation for this observation is that different species with more divergent enzymes (<50% aa identity) are responsible for coprostanol formation in human microbiomes, which would result in multiple clusters with lower sensitivity (but high specificity) contributing to the total number of coprostanol positive samples across the cohort. Under this assumption, if we defined the protein clusters with >0.3 sensitivity and >0.9 specificity for coprostanol as the most promising candidates for experimental validation, this stringent cutoff would still yield thousands of candidates (dataset 1 = 33 k, dataset 2 = 2 k), necessitating additional prioritization steps.

To identify protein clusters most likely to contain cholesterol-metabolizing enzymes, we integrated genomic information from the previously reported coprostanol-forming bacteria *Eubacterium coprostanoligenes*, *Bacteroides dorei*, *Lactobacillus sp.*, and *Bifidobacterium sp* (Freier et al., 1994, Gérard et al., 2007, Lye et al., 2010). First, we validated coprostanol formation by *E. coprostanoligenes* HL (ATCC 51222), a hog sewage lagoon isolate, which is the only publicly available strain displaying characteristics of the coprostanol-forming Eubacterium strains isolated in the 20th century (Figure S2A) (Freier et al., 1994). Although additional studies have suggested that *B. dorei*, *Lactobacillus*, and *Bifidobacterium* species can also perform this transformation, we did not observe coprostanol formation in a targeted screen of 17 different isolates of these species (Table S1); this activity is potentially a strain-specific adaptation and not a core function in these species (Freier et al., 1994, Gérard et al., 2007, Lye et al., 2010, Norin et al., 1991, Vatanen et al., 2019). As *E. coprostanoligenes* HL is the only available bacterial isolate capable of cholesterol metabolism, we sequenced and assembled a high-quality genome to search for enzymes involved in coprostanol formation (Parmentier and Eyssen, 1974). Excitingly, ~80% of *E. coprostanoligenes* proteins could be mapped to the *de novo* assembled clusters of homologous proteins (50% aa similarity), and 328 of these proteins showed >0.3 sensitivity and >0.9 specificity for coprostanol detection (Figure 2B). Only 12 such proteins were identified from all other reported coprostanol-forming species, making these species unlikely candidates for coprostanol formation in human microbiomes (Figure S1E). Altogether, by integrating screening results and genomic information, we greatly narrowed down candidate enzymes involved in cholesterol metabolism.

Since we hypothesized that the human gut microbial enzymes responsible for coprostanol formation would be related to the as-yet-undiscovered cholesterol-metabolizing enzyme(s) from *E. coprostanoligenes*, we next investigated how coprostanol formation is accomplished by this organism. Earlier studies using labeled cholesterol determined that coprostanol formation in this organism proceeds through an indirect reduction pathway involving the initial oxidation of cholesterol (1) to cholestenone (2) (Figure 1) (Björkhem and Gustafsson, 1971, Ren et al., 1996). To verify this finding, we tested the activity of *E. coprostanoligenes* lysates toward cholesterol under both aerobic and anaerobic conditions. We observed conversion of cholesterol to cholestenone and discovered that this reaction requires NADP^+^ and is oxygen independent (Figures S2B and S2C). The putative second step of this pathway, the formation of coprostanone (3) from cholestenone (2), did not occur in lysates, suggesting that either the assay conditions need further optimization or the putative enzyme becomes inactivated during cell lysis. Therefore, we prioritized finding the cholesterol-oxidizing enzyme.

Given that cholesterol oxidation in *E. coprostanoligenes* cell lysate was oxygen independent and the gut is an anaerobic environment, we reasoned that the well-studied oxygen-dependent cholesterol oxidases (PF09129) found in many *Streptomyces* species were unlikely to mediate this transformation (Kreit and Sampson, 2009). Accordingly, no homologs of any queried cholesterol oxidases were found in the genome of *E. coprostanoligenes* or our entire human microbiome gene catalog. The only characterized oxygen-independent enzymes capable of this reaction are the cholesterol dehydrogenases AcmA (PF01370) from the soil bacterium *Sterolibacterium denitrificans* and Rv1106c from *Mycobacterium tuberculosis* (Chiang et al., 2008, Yang et al., 2007). While no AcmA or Rv1106c homologs were found in the genome of *E. coprostanoligenes*, there were a significant number of homologs in the human microbiome gene catalog; however, none of the homologs had high specificity and sensitivity for coprostanol in our two metabolomics datasets (Figures 2C and S1E).

The final class of enzymes we considered was the hydroxysteroid dehydrogenases (HSDs), which belong to the short-chain dehydrogenase (SDR) enzyme family (PF00106). These enzymes are found in many gut microbes and can oxidize hydroxyl groups of bile acids to ketones in a NAD(P)^+^-dependent, oxygen-independent manner (Devlin and Fischbach, 2015). However, no characterized gut microbial HSDs are known to accept cholesterol as a substrate. Using 6 biochemically characterized bile acid-metabolizing HSDs from gut microbes (*E. lenta*, *R. gnavus*, and *E. coli*) as a query, we found 1,310 clusters of homologous proteins in the human microbiome gene catalog. Four of the HSD clusters contained homologs of *E. coprostanoligenes* proteins, including a cluster of 25 proteins associated with coprostanol formation in stool with 0.92 specificity and 0.68 sensitivity (Figures 2D and S1D; Table S2). In summary, our metagenome-wide search combined with metabolomics-, genome-, and enzyme-guided bioinformatics distilled 6M microbiome genes into 4 protein clusters that we prioritized for experimental validation.

### Biochemical Characterization of Putative Gut Microbial Cholesterol Dehydrogenases

**Figure 3:**
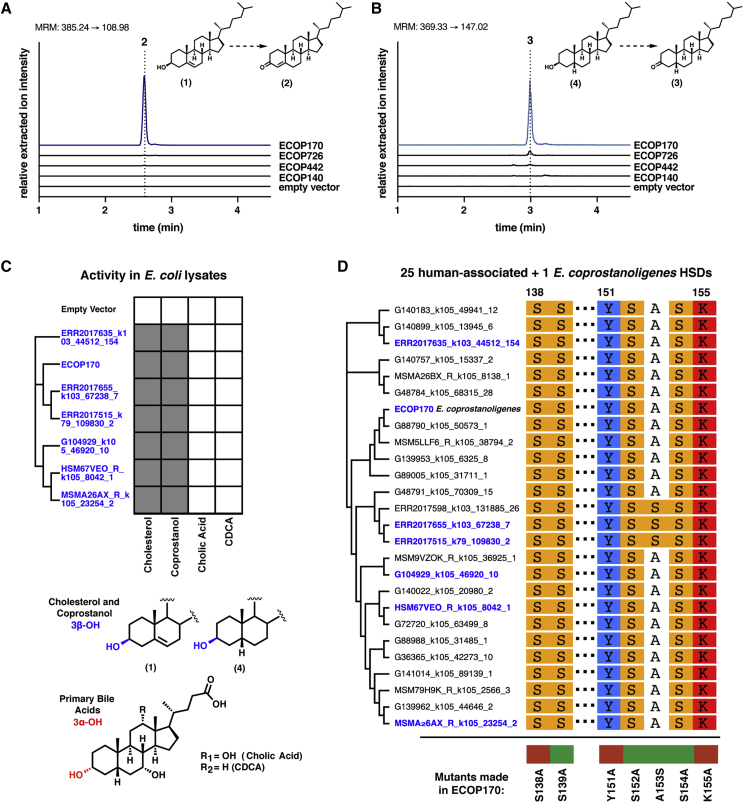
Uncharacterized 3β-Hydroxysteroid Dehydrogenase Enzymes from *E. coprostanoligenes* and Phylogenetically Related Human-Associated Bacteria Oxidize Cholesterol to Cholestenone (A and B) (A) A 3β-hydroxysteroid dehydrogenase (3β-HSD) enzyme from *E. coprostanoligenes*, ECOP170, converts cholesterol (1) to cholestenone (2) and (B) converts coprostanol (4) to coprostanone (3) in the presence of a mixture of 100 μM of NAD^+^ and 100 μM NADP^+^. (C) ECOP170 homologs from gut bacteria heterologously expressed in *E. coli* convert the 3β-OH groups (blue) of cholesterol and coprostanol (gray squares) to the corresponding ketones (2 and 3, respectively) but were not able to convert primary bile acids, which have 3α-OH groups (red), to the corresponding keto bile acids (white squares). We considered the detection of any of the desired products after overnight incubation in an assay condition to be metabolism. (D) A multiple sequence alignment of the 25 human-associated cholesterol dehydrogenase homologs and ECOP170 showing the conserved active site residues S138, Y151, and K155. Mutation of any of these residues to alanine in ECOP170 completely abolishes activity (red), whereas proteins with mutations in neighboring residues retain activity (green). Cholesterol dehydrogenases highlighted in blue are confirmed biochemically to oxidize cholesterol. See also Figures S2–S4 and Tables S2 and S3.

To test whether proteins from these prioritized clusters could oxidize cholesterol (1) to cholestenone (2), we expressed each of the four putative HSDs encoded by *E. coprostanoligenes* in *E. coli* and evaluated the reactivity of cell lysates toward cholesterol (Figure 3A). ECOP170 (WP_078769004.1), the *E. coprostanoligenes* HSD with the highest specificity toward coprostanol in stool metabolomes, oxidized cholesterol to cholestenone, completing the first step in cholesterol metabolism. ECOP170 also catalyzed the oxidation of coprostanol (4) to coprostanone (3), but did not transform the primary bile acids cholic acid and chenodeoxycholic acid, which have 3α-OH groups rather than 3β-OH groups, suggesting it may be specifically responsible for both the first (oxidation of cholesterol to cholestenone) and last (reduction of coprostanone to coprostanol) steps of this pathway (Figures 3B, 3C, S4B, and S4C).

As it is currently impossible to access a targeted knockout of ECOP170 in *E. coprostanoligenes* due to lack of tools for genetic manipulation, we confirmed ECOP170’s role in cholesterol metabolism by measuring its expression levels in active *E. coprostanoligenes* cultures and matching its cofactor preferences to the activity in *E. coprostanoligenes* lysates. To confirm that ECOP170 was expressed by *E. coprostanoligenes* under cholesterol-metabolizing conditions, levels of transcripts encoding all four HSDs were measured when *E. coprostanoligenes* was cultivated with and without cholesterol (Figure S2D; Table S3). Of these four genes, ECOP170 had the largest increase in expression in medium containing cholesterol compared with medium lacking cholesterol after 2 days of growth (28.9-fold increase), suggesting it is highly induced under conditions where cholesterol is present. In order to determine if ECOP170’s cofactor preference matched the activity observed in *E. coprostanoligenes* lysate, we purified N-terminal His_6_-tagged ECOP170 and assayed it for activity (Figure S2E). ECOP170 showed a strict dependence on NADP^+^ and not NAD^+^, directly matching the activity we observed in *E. coprostanoligenes* lysate (Figures S2C and S2F). Together these data suggest that ECOP170 is the enzyme responsible for the metabolism of cholesterol to cholestenone in *E. coprostanoligenes*. With this information, we named ECOP170 *ismA* to indicate its substrates and the stage at which it acts during coprostanol formation from cholesterol.

Having determined that IsmA is a cholesterol dehydrogenase, we returned to the human microbiome gene catalog to examine the other proteins in the IsmA-containing cluster (Table S2). While we initially used a 50% aa identity cutoff for assembling our protein clusters, we also wanted to determine the specificity and sensitivity of each individual protein in the IsmA-containing cluster for the presence of coprostanol. Although each protein showed near-perfect specificity, they had much lower sensitivity than the IsmA-containing cluster, demonstrating the utility of grouping protein sequences for prioritization at the onset (Figure S1F). Of the 25 protein sequences in that cluster, only 10 were previously deposited in the NCBI database, all of which were assigned to co-abundant gene groups (CAGs), i.e., microbial species that lack cultured representatives and were only identified in gut microbiome assemblies (Figure S3) (Nielsen et al., 2014). Furthermore, neither these 10 nor the other 15 proteins within the cluster could be assigned to isolates in the NCBI database or in recently described gut microbiota strain collections (Forster et al., 2019, Zou et al., 2019).

Since there are currently no available human gut microbial isolates encoding any of the IsmA homologs, we selected six homologs of diverse sequence that were prevalent in the studied datasets for heterologous expression in *E. coli* and *in vitro* biochemical characterization. All six IsmA homologs examined oxidized both cholesterol to cholestenone and coprostanol to coprostanone in *E. coli* lysates (Figures 3C and S4). A multiple sequence alignment of the 25 protein sequences within the IsmA-containing cluster revealed the strictly conserved catalytic triad of Ser-Tyr-Lys required for HSD activity (Figures 3D and S2G). Mutating any of these three amino acids in *Eubacterium coprostanoligenes* IsmA led to complete loss of cholesterol-oxidizing activity in lysates (Figures 3D, S2H, and S2I). These data suggest that the homologs of *Eubacterium coprostanoligenes* IsmA found in uncultivated human gut bacteria are also cholesterol dehydrogenases involved in the first and last steps (Figure 1) of coprostanol metabolism in human gut microbiotas.

### Gut Microbial Cholesterol Dehydrogenases Are Encoded by a Clade of Prevalent but Uncultured Bacteria Related to Cluster IV Clostridium

Because the 25 *ismA* genes found in human microbiomes could not be mapped back to any publicly available isolate genome, and 10 of those genes were associated with metagenomic species (Nielsen et al., 2014), we wanted to determine if the other *ismA* genes could also be assigned to uncultivated microbial species. To do this, we binned the assembled human gut metagenomes into metagenomic species (MSPs) using MSPminer and searched these species for the 25 *ismA* genes (Plaza Oñate et al., 2019). Using this approach, 19 of the 25 homologs were successfully assigned to individual MSPs. Similarity based taxonomic annotation of these MSPs at the species level using a comprehensive collection of microbial isolates was unsuccessful, confirming that these cholesterol-metabolizing human gut bacteria have not been previously characterized.

**Figure 4:**
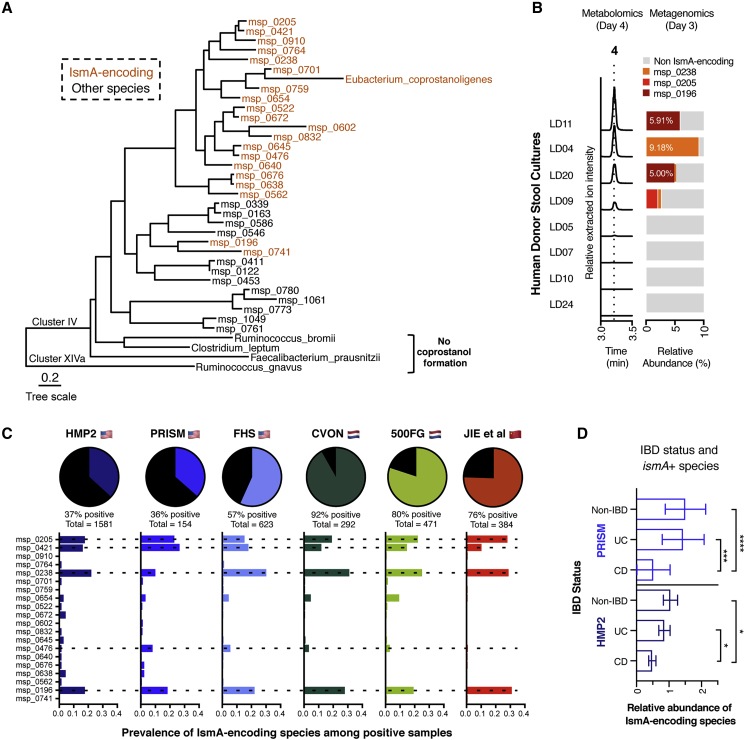
Cholesterol Dehydrogenase-Encoding Gut Bacteria Are Uncultured Members of Cluster IV Clostridium and Are Prevalent Across Geographically Diverse Human Populations (A) 20 different MSPs containing *ismA* genes could be identified in human gut microbiome datasets. Phylogenetic tree was generated using PhyloPhlAn and includes all IsmA-encoding MSPs as well as species in the direct neighborhood or marker species for Clostridium cluster IV and cluster XIVa. (B) *Ex vivo* conversion of cholesterol to coprostanol by human fecal samples. Coprostanol formation occurred in 4 of the 8 samples cultured in basal cholesterol medium, with all 4 metabolizing samples containing at least one of the IsmA-encoding species identified at day 3. (C) Proportion of microbiome samples within each respective cohort that contains at least one IsmA-encoding species. IsmA-encoding species msp_0205, msp_0421, msp_0238, and msp_0196 are the most abundant across all of the populations examined. Dotted lines show species whose IsmA proteins have been shown to metabolize cholesterol *in vitro*. (D) Relative abundance levels of IsmA-encoding species present in the gut microbiome when stratified by disease state (HMP2: total, n = 1,581; non-IBD, n = 411, avg rel. ab. = 1.047; UC, n = 437, avg rel. ab. = 0.8613; CD, n = 733, avg rel. ab. = 0.4864; non-IBD versus UC p = 0.72; non-IBD versus CD p = 0.009; UC versus CD p = 0.009; PRISM: total, n = 154; non-IBD, n = 34, avg rel. ab. = 1.51; UC, n = 52, avg rel. ab. = 1.437; CD, n = 68, avg rel. ab. = 0.525; non-IBD versus UC p = 0.14; non-IBD versus CD p = 1.33e−6; UC versus CD p = 6.30e−4). p values were determined by a Wald test for PRISM (a linear model) and a Satterthwaite's method for HMP2 (a mixed linear model with random effect for subjects) and corrected for multiple comparisons with Benjamini-Hochberg method. The center bar represents the mean and error bars representing 95% CIs. See also Figure S5 and Table S4.

To aid in taxonomic annotation, we evaluated the phylogenetic relationship of all detected MSPs to known microbial isolates using a set of single-copy marker genes (PhyloPhlAn; Segata et al., 2013). In the bacterial tree of life, the IsmA-encoding MSPs and *E. coprostanoligenes* form a coherent clade that is situated in the phylogenetic neighborhood of Clostridium cluster IV. Cluster IV contains species such as *Faecalibacterium prausnitzii*, *Clostridium leptum*, and *Ruminococcus bromii* (Figure 4A) that possess metabolic capabilities linked to host health, including short-chain fatty acid production (Lopetuso et al., 2013). In the direct neighborhood of IsmA-encoding MSPs, we observed 9 additional MSPs; upon further examination of their high-quality draft genome assemblies, two (msp_0910 and msp_0832) were also confirmed to encode *ismA* genes and were included in all further analyses. The *ismA* gene from msp_0832 was missed in the original assembly of the non-redundant gene catalog, which suggests that additional examples of *ismA* genes and IsmA-encoding species might be uncovered by employing different assembly techniques.

At least one high-quality draft genome for most of the IsmA-encoding MSPs was generated (completeness > 90%, contamination < 5%; Parks et al., 2015), and genome-wide similarity comparison confirmed that these MSPs are different species with a maximum average nucleotide identity of 88% (Figure S5E; Table S4) (Jain et al., 2018). We then compared the high-quality genomes of IsmA-encoding MSPs with previously recovered novel MSPs from human gut metagenomes and confirmed that all but two of these species were also cataloged in earlier efforts (Figure S5B; Table S4) (Almeida et al., 2019, Nielsen et al., 2014, Pasolli et al., 2019, Plaza Oñate et al., 2019). To expand our search to other microbiome datasets not included in our initial assembly, we queried metagenomic species from Pasolli et al. and Almeida et al. for IsmA proteins, identifying 14 additional IsmA proteins (50% aa identity cutoff) and their corresponding metagenomic species (Figure S5D; Table S2). Notably, when commonly used reference-based microbiome analysis software (MetaPhlAn2, MetaPhlAn3, and mOTUs_v2) were used to analyze IsmA-encoding genomes, less than a quarter of the species could be assigned, illustrating the current limitation of the reference-based microbiome analysis (Table S4) (Milanese et al., 2019, Truong et al., 2015).

Given the lack of coprostanol-forming human gut isolates, we wanted to test whether microbial communities containing IsmA-encoding species could generate coprostanol *ex vivo*. To accomplish this, we cultured stool samples from eight healthy donors anaerobically in a cholesterol-containing medium for 4 days. Metagenomic sequencing was performed on day 3, and levels of cholesterol, cholestenone, and coprostanol were measured on days 2 and 4. IsmA-encoding bacteria could be detected in the four samples where coprostanol was produced on day 4, whereas samples without coprostanol lacked IsmA-encoding bacteria, further connecting the presence of these species in complex microbial communities with cholesterol metabolism (Figure 4B). While complete conversion of cholesterol to coprostanol was never observed, similar levels of coprostanol were formed in the stool cultures as in axenic cultures of *E. coprostanoligenes*, suggesting there may be a limit to this metabolic transformation under these specific culture conditions (Figure S2A).

To understand the distribution of the 20 IsmA*-*encoding species in the human gut, we stringently mapped the metagenomic datasets against the non-redundant gene catalog to calculate the prevalence and relative abundance of the individual species in each dataset. Across the six cohorts used to make the initial assembly, IsmA-encoding species had an average relative abundance of 1.4% (Figure S5B), while the percentage of samples containing an IsmA-encoding species varied from 37% of samples in the human microbiome project 2 (HMP2) cohort to 92% of samples in the CVON cohort (Figure 4C). The two cohorts with the lowest percentage of encoders were PRISM and HMP2, both of which contain significant numbers of samples from inflammatory bowel disease (IBD) patients. In these two IBD cohorts, Crohn’s disease was significantly associated with decreased abundance of IsmA-encoding species, suggesting that these bacteria may be sensitive to terminal ileum inflammation (Figure 4D) (Santoru et al., 2017, Sundqvist et al., 2019). The IsmA homologs we characterized *in vitro* were encoded by the most prevalent MSPs found in all six cohorts (Figure 4C); msp_0205, msp_0238, and msp_0196 were also present in the coprostanol-producing stool cultures (Figure 4B). Together, these data support the idea that IsmA-encoding bacteria are prevalent constituents of the human gut microbiome where they convert cholesterol to coprostanol.

### The Presence of IsmA-Encoding Bacteria Is Associated with Lower Stool Cholesterol and Elevated Levels of Stool Cholestenone and Coprostanol

**Figure 5:**
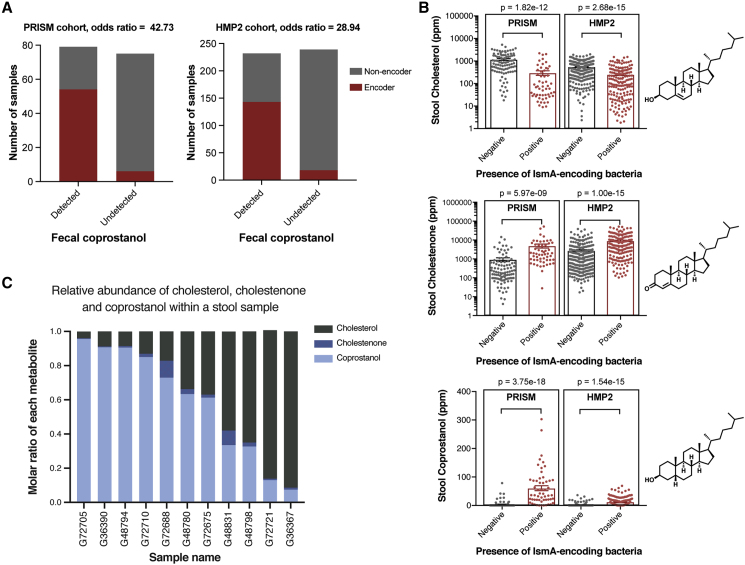
Fecal Coprostanol Formation Is Correlated to the Presence of Cholesterol Dehydrogenases in Gut Microbiomes (A) Two independent human cohorts with paired fecal metagenomics and metabolomics were used to investigate the association between IsmA-encoding species and coprostanol formation. The presence of IsmA-encoding bacteria in the gut microbiome is highly correlated to the presence of fecal coprostanol (detected). Odds ratios for PRISM and HMP2 cohorts are 42.73 (95% CI: 11.28; 283.54) and 28.94 (95% CI: 13.64; 61.41), respectively. (B) Stool samples from patients with *ismA+* species in their microbiotas have lower stool cholesterol (1), and higher cholestenone (2) and coprostanol (4) as determined by untargeted fecal metabolomics. Each point represents an independent sample with the center bar representing the mean and error bars representing SEM (PRISM: *ismA+* species negative samples n = 99, positive samples n = 55, HMP2: *ismA+* species negative samples n = 302, positive samples n = 169). Analysis was performed using linear (cholesterol and cholestenone) and logistic (coprostanol) regressions for PRISM and mixed effect linear (cholesterol and cholestenone) and logistic (coprostanol) models to account for repeated measures in HMP2, including the following as covariates in all models: age, gender, antibiotic usage (yes/no) and disease status (non-IBD, CD, or UC). See STAR Methods for details. (C) Molar ratios of cholesterol (gray), cholestenone (dark blue), and coprostanol (light blue) are shown for 11 coprostanol-positive stool samples from the PRISM cohort as measured by targeted metabolomics. Across the samples, molar ratios for coprostanol vary from 0.956 in sample 8,982 to 0.074 in sample 8,573. See also Figure S6 and Table S5.

With a census of IsmA-encoding bacteria completed, we wanted to evaluate the extent to which the presence of these bacteria in complex microbial communities is associated with coprostanol formation *in vivo* (Figure 5A). Returning to the two independent cohorts with paired metagenomic and metabolomic data, we categorized samples as either coprostanol positive (converters) or coprostanol negative (non-converters), as determined by the presence of coprostanol in their fecal metabolomes (see STAR Methods for details) (Franzosa et al., 2019, Lloyd-Price et al., 2019). In both cohorts, converter samples were strongly enriched in IsmA-encoding species (PRISM: OR = 42.73 [95% confidence interval, CI: 11.28, 283.54]; HMP2: OR = 28.94 [95% CI: 13.64, 61.41]) (Figure 5B; Table S5). This supports the hypothesis that the presence of IsmA-encoding bacteria within a gut microbiome confers the community with the ability to metabolize cholesterol to coprostanol. Interestingly, we also observed that in a subset of microbial communities without a known IsmA-encoding microbe, coprostanol was still present (Figure 5B). This observation might be explained by the limit of detection associated with metagenomic sequencing, especially with low abundance microbes, or the possibility that other, more distantly related microbes and their cholesterol dehydrogenases can also perform this reaction.

In addition to their association with coprostanol, we also evaluated whether the presence of IsmA-encoding species correlated with changes in levels of fecal cholesterol and other pathway intermediates identified in the untargeted metabolomics datasets. Strikingly, we observed a 75% and 55% reduction in stool cholesterol in IsmA-encoders versus non-encoders in the PRISM and HMP2 cohorts, respectively (Figure 5C; Table S5). We observed a corresponding increase in levels of stool cholestenone, with IsmA-encoders having 5.4- and 3.3-fold increases in cholestenone compared with non-encoders in the two cohorts (Figure 5C; Table S5). These shifts in metabolite levels and their co-occurrence with *ismA* homologs are consistent with our hypothesis that IsmA-encoding gut bacteria deplete intestinal cholesterol through oxidation of cholesterol to cholestenone, an on-pathway intermediate to coprostanol formation.

In order to determine the extent of cholesterol metabolism in stool samples where coprostanol is being formed, we re-ran a subset of 26 samples from the PRISM cohort using a quantitative liquid chromatography-mass spectrometry (LC-MS) method. Our concentration values for the three metabolites measured (cholesterol, cholestenone, and coprostanol) correlated well with their respective relative abundances determined by previous metabolomic methods in the same samples, validating the quantitative nature of the fecal metabolomics data for our metabolites of interest (Figure S6A). Using the determined concentrations of each compound within a stool sample, we calculated the relative proportion of coprostanol, cholestenone, and cholesterol in each sample (Figures 5C, S6B, and S6C). In the 11 coprostanol-containing human stool samples, the percentage of coprostanol to total measured cholesterol and cholesterol metabolites (cholesterol, cholestenone, and coprostanol) ranged from 7.4% to 95.6%, indicating a wide range of activity for this metabolism *in vivo* (Figure 5C). Coprostanol comprised greater than 50% of the cholesterol metabolites in 7 of the 11 samples; in 3 of these 7 samples, coprostanol accounted for greater than 90% of the cholesterol metabolites. These data suggest that in some people, this pathway has the potential to convert most of the intestinal cholesterol to coprostanol *in vivo*.

### The Presence of IsmA-Encoding Bacteria Is Associated with Lower Levels of Serum Cholesterol

Because IsmA-encoding bacteria are highly associated with both coprostanol formation in human stool samples and decreased fecal cholesterol levels, we next examined whether the presence of these coprostanol-forming bacteria is associated with variation in serum lipid levels in human populations, specifically high-density lipoprotein (HDL)-C, low-density lipoprotein (LDL)-C, and total cholesterol (TC). As a comprehensive approach, we used three studies (Framingham heart study [FHS], CVON, and Jie et al.) with paired stool metagenomics and serum cholesterol measurements, comprised of participants from three different countries (USA, the Netherlands, and China, respectively) (Table S6). CVON (n = 292) and Jie et al. (n = 384) were previously published, while stool metagenomics data from 623 subjects in the FHS cohort were generated by our lab to aid in answering this question. The chosen studies also included participants with prevalent CVD. Participants whose microbiome harbored any of the identified IsmA-encoding bacteria were classified as encoders, while those without these species were considered non-encoders.

**Figure 6:**
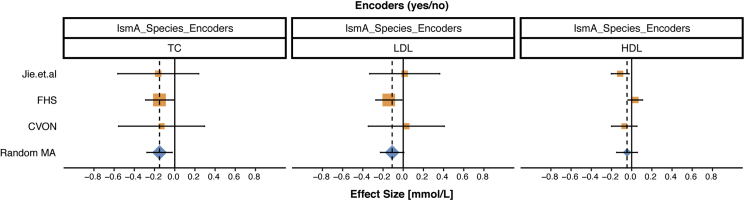
Meta-Analysis Reveals an Association of Total Cholesterol Levels with Encoder Status in CVD Cohorts Three studies were included in random effects meta-analysis (highlighted in orange and annotated on y axis). The changes in HDL-C, LDL-C, and TC levels (mmol/L) between encoders and non-encoders are represented for each study by the center of the square (95% CI presented by respective horizontal black lines). The combined results of the meta-analysis are represented by blue diamonds with point estimate presented by vertical diamond points and dashed line, whereas respective 95% CI is presented by the horizontal line in the diamond's center. Solid black line represents the null effect. Studies on the right of this line (i.e., positive values on the x axis) have a higher level of serum lipids in encoders compared with non-encoders; studies on the left (i.e., negative values on the x axis) have lower levels of serum lipids in encoders than in non-encoders. In a meta-analysis of three studies, we observed 0.15-mmol/L lower level of TC in encoders than in non-encoders (95% CI: −0.27, −0.03). Meta-analysis results for HDL-C and LDL-C were not statistically significant. See also Table S6 for full results.

In a meta-analysis of these studies, while no statistically significant effects were observed for either LDL-C or HDL-C, we did observe a pooled difference of −0.15 mmol/L in TC (95% CI: −0.27, −0.03) between encoders and non-encoders (Figure 6; Table S6), with directional consistency across all three studies and low between-study heterogeneity (I^2^ = 0). To put the magnitude of the observed decrease in serum cholesterol levels into perspective, the effect sizes of the IsmA-encoding bacteria (0.15 mmol/L for TC) slightly exceed the largest effects of lipid-associated host genes, such as *HMGCR* (0.068 SD units per allele for TC, which corresponds to 0.063 mmol/L for the FHS study; see STAR Methods for details) or *PCSK9* (0.054 SD units per allele for TC, which corresponds to 0.050 mmol/L per allele for TC for the FHS study) (Willer et al., 2013). Since the presence of these IsmA-encoding bacteria significantly correlates with a biologically meaningful decrease in total serum cholesterol, it suggests that cholesterol metabolism by the human gut microbiota decreases host cholesterol levels.

## Discussion

The idea that gut bacterial metabolism of cholesterol to coprostanol may lower serum cholesterol levels was proposed over 100 years ago, yet relatively few studies have investigated this connection. The paucity of information about this process is especially striking considering the abundance of information regarding other gut microbial metabolic activities, such as secondary bile acid formation and short-chain fatty acid biosynthesis. Efforts to understand the biological implications of gut bacterial cholesterol metabolism have been hindered by the difficulty of culturing microbes responsible for this activity in humans and a lack of knowledge regarding the biochemical and genetic basis for this metabolic process (Ooi and Liong, 2010). By combining large-scale sequencing efforts, reference-free microbiome analysis, and a suite of *in vitro* biochemical and culture-based assays, we have identified and characterized gut bacterial enzymes responsible for the first and last step in coprostanol formation. Our work shows that the majority of coprostanol formation in diverse human populations can be attributed to a clade of highly prevalent, IsmA-encoding bacterial species. These species were previously uncharacterized and currently remain uncultured, potentially explaining the past difficulties in studying this metabolic pathway. We observed that the presence of coprostanol-forming bacteria in stool samples is associated with lower levels of fecal cholesterol, providing a plausible mechanism by which these bacteria may decrease host serum cholesterol levels. This concept is supported by the results of our meta-analysis of three geographically diverse human cohorts, which shows that subjects with coprostanol-forming microbes have lower total serum cholesterol.

In addition to the observed correlation with changes in serum lipid levels, the presence of these IsmA-encoding bacteria is associated with highly elevated levels of intestinal cholestenone and coprostanol. This phenomenon merits further investigation, as little is known about the effects of either of these molecules on the host. Metabolites with similar chemical structures, such as bile acids, have large effects on host metabolism and immune regulation, so it is plausible that both cholestenone and coprostanol may influence host biology (Sinha et al., 2020, Song et al., 2020, Yao et al., 2018). Our newfound understanding of which gut bacteria perform this reaction will guide analysis of gut metagenomic datasets to identify additional biological phenomena in which intestinal cholesterol metabolism plays a role. More generally, this work underscores the critical need to link gut microbial metabolic activities to organisms, genes, and enzymes to fully understand metabolic interactions with the human host (Maini Rekdal et al., 2019).

The effect sizes of the presence of IsmA-encoding bacteria on serum cholesterol is on par with those associated with differences in human genes, pointing to a potentially protective role for these bacteria in CVD, as is observed for variants of human genes. Since our current cohorts are statistically underpowered to assess the associations between CVD risk and gut microbiome composition, large-scale prospective studies are most likely required to explore this link. However, since targeting these human genes with therapeutic interventions produces larger effect sizes (for statins targeting HMGCR: on average 1.20 mmol/L on TC), it is possible that modulating the activity of this microbial pathway may lead to similar increases in effect size and additional therapeutic benefit (Law et al., 2003). By introducing cholesterol-metabolizing gut bacteria into human gut microbiotas, or by increasing their abundance with prebiotics, it may be possible to achieve targeted effects on host serum cholesterol, a strategy that has already shown promise in influencing other areas of human metabolism (Holscher, 2017, Kurtz et al., 2019).

The discovery of enzymes involved in cholesterol metabolism from the gut microbiome was accomplished using a multi-disciplinary strategy integrating high-throughput readouts (*de novo* gene assembly and metabolomics) and biochemical knowledge. This same discovery strategy can also be applied to other pathways/metabolites of interest and will enable further identification and characterization of enzymes involved in biological processes contributed by the microbiota, regardless of whether the microbe responsible for this metabolism is known. This is especially important for microbiome studies as computational methods continue to reveal uncharacterized microbes and enzymes that exist in microbial communities across the globe (Almeida et al., 2019, Pasolli et al., 2019). While characterizing “microbial dark matter” still presents significant challenges, combining bioinformatic and biochemical approaches has the potential to grant access to this largely untapped source of biologically relevant metabolic transformations (Marcy et al., 2007, Rinke et al., 2013).

## STAR★Methods

### Key Resources Table

**Table:** 

REAGENT or RESOURCE	SOURCE	IDENTIFIER
**Bacterial and Virus Strains**		

*E. coprostanoligenes*	American Type Culture Collection	ATCC51222
*E.Coli*	ThermoFisher	BL2- EC0114

**Biological Samples**		

Stool culture	This paper	N/A

**Chemicals, Peptides, and Recombinant Proteins**		

β-Nicotinamide adenine dinucleotide hydrate	Sigma	N6522-1G
β-NADH phosphate disodium salt	Sigma	10128040001
Coprostan-3-ol	Sigma	C7578-50MG
L-α-Phosphatidylcholine	Sigma	P3644-25G
Cholesterol	Sigma	C3045-5G
Sodium thioglycolate	Sigma	T0632-25G
Calcium Chloride, Dihydrate, Molecular Biology Grade - CAS 10035-04-8 – Calbiochem	Sigma	C5670-100g
Yeast Extract	Sigma	Y1625-250G
(+)-4-Cholesten-3-one (cholestenone)	Sigma	188174-10G
BD Difco Agar, Technical	Fisher Scientific	DF0812-17-9
cOmplete, EDTA-free Protease Inhibitor Cocktail	Sigma	11873580001
Lysonase Bioprocessing Reagent	Sigma	71230

**Critical Commercial Assays**		

DNeasy PowerSoil Kit	Qiagen	12888
High-Capacity cDNA Reverse Transcription Kit	Applied Biosystems	4368813
iTaq Universal SYBR Green Supermix	BioRad	1725121

**Deposited Data**		

Framingham Heart Study metagenomics data	This paper	BioProject PRJNA559860
*Eubacterium coprostanoligenes* genome	This paper	BioProject PRJNA559861
Stool Culture sequencing data	This paper	BioProject PRJNA559861

**Oligonucleotides**		

Primers for qPCR of putative cholesterol oxidoreductases in *E. coprostanoligenes*, see Table S3	This paper	N/A
Primers for cloning putative cholesterol oxidoreductases into the pET28 vector, see Table S3	This paper	N/A
Primers for site directed mutagenesis of ECOP170, see Table S3	This paper	N/A

**Recombinant DNA**		

pET28 plasmid	N/A	N/A
Geneblocks for the cholesterol oxidoreductases from human-associated bacteria, see Table S3	Genewiz	N/A

**Software and Algorithms**		

Trim_Galore! (v0.4.4 )	Babraham Bioinformatics	https://www.bioinformatics.babraham.ac.uk/projects/trim_galore/
Trimmomatic (v0.36)	Bolger et al., 2014	http://www.usadellab.org/cms/?page=trimmomatic
KneadData (v0.7.2)	Huttenhower lab	http://huttenhower.sph.harvard.edu/kneaddata
MegaHIT (v1.1.4)	Li et al., 2015	https://github.com/voutcn/megahit
Prodigal (v2.6.3)	Hyatt et al. 2010	https://github.com/hyattpd/prodigal/releases/
SPAdes (v3.9.0)	Bankevich et al. 2012	https://github.com/ablab/spades/releases
BWA (v0.7.17)	Li and Durbin, 2009	http://bio-bwa.sourceforge.net/
SAMTools (v1.8)	Li and Durbin, 2009	http://www.htslib.org/
CD-HIT (v4.7)	Fu et al. 2012	http://weizhongli-lab.org/cd-hit/
CheckM (v1.0.13)	Parks et al. 2015	https://github.com/Ecogenomics/CheckM/releases
PhyloPhlAn (v0.99)	Segata et al., 2013	https://huttenhower.sph.harvard.edu/phylophlan/
FastANI (v1.2)	Jain et al. 2018	N/A
EggNOG Mapper (v1.0.3)	Huerta-Cepas et al., 2016, Huerta-Cepas et al., 2019	http://eggnog-mapper.embl.de/
USEARCH (v8.1)	Edgar, 2010	https://www.drive5.com/usearch/
BLAST+ (v2.6.0+)	NCBI	https://blast.ncbi.nlm.nih.gov/Blast.cgi?PAGE_TYPE=BlastDocs&DOC_TYPE=Download
MSPminer	Plaza Oñate et al., 2019	https://www.enterome.com/downloads/
R (v3.6.1)	packages for analytical part: *stats*, *nlme*, *lme4*, *meta*	https://www.r-project.org/

### Resource Availability

#### Lead Contact

Further information and requests for resources and reagents should be directed to and will be fulfilled by the Lead Contact, Ramnik J. Xavier (rxavier@broadinstitute.org).

#### Materials Availability

All plasmids generated in this study are available upon request from the Lead Contact.

#### Data and Code Availability

PRISM and HMP2 metabolomics data (accession number PR000677 and PR000639 respectively) are available at the NIH Common Fund’s Metabolomics Data Repository and Coordinating Center (supported by NIH grant, U01-DK097430): Metabolomics Workbench (http://www.metabolomicsworkbench.org). Framingham Heart Study metagenomics data is available in the Sequence Read Archive (https://www.ncbi.nlm.nih.gov/sra) (SRA): PRJNA559860. Stool culture sequencing data can be found in SRA: PRJNA559861.

### Experimental Model and Subject Details

#### Bacterial Strains

*E. coprostanoligenes* ATCC51222 was obtained from the American Type Culture Collection. *E. coprostanoligenes* and stool cultures were grown in basal cholesterol medium (BCM), which contained (per liter) 10 g of casitone (Difco Laboratories, Detroit, Mich.), 10 g of yeast extract, 2 g of cholesterol, 1 g of lecithin, 0.5 g of sodium thioglycolate, 1 g of calcium chloride dihydrate, and 1 mg of resazurin. *E. coprostanoligenes* was grown on modified lecithin agar medium (MLA) plates, which was prepared as described by Freier et al. (1994).

Cultures were grown and handled in an anaerobic chamber (Coy Laboratory Products) with an atmosphere of 20% CO_2_, 5% H_2_, and 75% N_2_ at 37°C.

#### Human Subjects

The Framingham Heart Study (FHS) is an observational longitudinal epidemiological investigation of the development of disease as it evolves in a community-based population sample. The design involves serial examination of all Framingham cohorts. The examinations include laboratory testing, physical examination, and interviews. For this study, participants were part of the Generation 3/Omni 2 Cohorts who agreed to participate in the microbiome analysis at exam 3 (2016-2019).

The Generation 3 cohort was initially recruited from 2002-2005 and consists of adult men and women who were at least 20 years-old by the close of Generation 3 Exam 1, and who have at least one parent in the FHS Offspring cohort. The omni 2 cohort participants represent an ethnically diverse group who were recruited from 2003-2005. All participants who came in for exam 3 were informed of the microbiome study. Actual participants were those who returned a sample kit after the visit.

The study protocol was approved by the Massachusetts General Hospital/Partners Human Research Committee and the Institutional Review Board of the Boston University Medical Center. All experiments adhered to the regulations of these review boards. All study procedures were performed in compliance with all relevant ethical regulations. Each participant signed an informed consent prior to participation.

### Method Details

#### Cloning and Expression of Candidate Cholesterol Dehydrogenase Genes

Candidate cholesterol dehydrogenase genes were amplified from genomic DNA (for *E. coprostanoligenes*) and cloned into pET28b. DNA was extracted from stool samples (DNeasy PowerSoil Kit, Qiagen) (for the *ismA* genes from msp_0238, msp_0205, msp_0421, Table S3) or purchased from Genewiz (Ordered sequences are listed in Table S2). Site-directed mutagenesis of residues in ECOP170 was accomplished using primers listed in Table S3. PCR reactions were performed with Phusion High Fidelity polymerase, and PCR products were purified (Zymoclean gel DNA recovery kit, Zymo research). The resulting gene products were assembled into pET28b using Gibson assembly and transformed into Stellar Competent Cells. The identities of the constructs were confirmed with DNA sequencing and transformed into *E. coli* BL21 strains for expression. All constructs were grown in LB with kanamycin (50 μg/mL) with the exception of the strain expressing the homolog from CAG:180 which required growth in TB for protein expression. All constructs were induced at an OD_600_ of 0.5–0.6 with 500 μM isopropyl β-D-1-thiogalactopyranoside, and the induced cells were incubated at 20°C for 20 h.

#### Lysate Experiments for Cholesterol Dehydrogenase Activity in *E. coli*

500 mL of a culture of *E. coli* BL21 expressing one of the cholesterol dehydrogenase homologs were pelleted by centrifugation (20 min at 7,000*g* and 4°C), resuspended in 10 mL of ice-cold phosphate-buffered saline containing one cOmplete Protease Inhibitor cocktail tablet (Roche Diagnostics) and lysed by a cell disruptor (EmulsiFlex-C3, Avestin). Cell debris was removed by ultracentrifugation (30 min at 20,000*g* and 4°C). Protein expression was confirmed by SDS-PAGE analysis using 4–20% Mini-PROTEAN TGX gels (Bio-Rad Laboratories). Gels were stained with Coomassie Blue for visualization. The clarified supernatant was used directly in the cell lysate assay described below. Cholesterol or coprostanol (5 μL of a 10 mM solution of cholesterol or coprostanol in methanol) was added to 500 μL of clarified supernatant with 100 μM of NADP^+^ and NAD^+^. After incubation at 37°C for 12 h, the reaction mixtures were frozen until being analyzed using LC-MS.

#### Lysate Experiments with *E. coprostanoligenes*

*Eubacterium* coprostanoligenes was grown under anaerobic conditions for 48 hours in basal cholesterol medium (BCM) without cholesterol in order to remove background cholesterol metabolites. 250 mL of a culture of *E. coli* BL21 expressing one of the cholesterol dehydrogenase homologs were pelleted by centrifugation anaerobically (20 min at 7,000*g* and 4°C), resuspended in 10 mL of ice-cold lysis buffer (50mM Hepes, 300mM NaCl, pH=7.5) containing one cOmplete Protease Inhibitor cocktail tablet and Lysonase Bioprocessing Reagent (Millipore Sigma) at the recommended concentration. Lysis was accomplished by sonication under anaerobic conditions and clarified by ultracentrifugation (30 min at 20,000*g*, 4°C, anaerobic conditions). Experiments were all set up under anaerobic conditions in an anaerobic chamber. For experiments under oxygen conditions, reactions were brought out of the chamber and opened to air.

#### qPCR of *E. coprostanoligenes*

Total RNA was purified by chloroform-phenol extraction from cell pellets of triplicate cultures of *E. coprostanoligenes* grown in BCM with or without cholesterol for 48 h. RNA was DNase treated, and cDNA was prepared using the High-Capacity cDNA Reverse Transcription Kit (Applied Biosystems). Transcripts of interest were quantified by real-time PCR carried out using iTaq Universal SYBR Green Supermix (Bio-Rad). All qPCRs were normalized to 16S rRNA gene expression. Primers used are listed in Table S3.

#### Purification of N-His Terminal Tagged ECOP170

Proteins were overexpressed using the procedure described above. Cells from 200 mL of culture were pelleted by centrifugation, resuspended in 10 mL of ice-cold lysis buffer (300 mM NaCl, 10 mM imidazole, 50 mM HEPES, pH 7.5) containing one cOmplete Protease Inhibitor cocktail tablet, and lysed by 4 min of continuous passage through a cell disruptor (EmulsiFlex-C3, Avestin) at 15,000 lbs per square inch. Cell debris was removed by ultracentrifugation (20 min at 20,000 X g and 4°C), and the cell-free extract was applied to 0.5 mL of HisPur Ni-NTA Resin (Thermo Scientific) pre-equilibrated with lysis buffer by gentle rocking at 4°C for 2 h. Non-absorbed materials and weakly bound proteins were removed by washing the column with 2 × 25 mL of wash buffer (300 mM NaCl, 20 mM imidazole, 50 mM HEPES, pH 7.5). His_6_-tagged protein was eluted with 5 mL of elution buffer (300 mM NaCl, 200 mM imidazole, 50 mM HEPES, pH 7.5). After SDS-PAGE analysis, eluent containing pure protein was dialyzed (Spectra/Por Dialysis Membrane, 6 – 8 kDa molecular weight cutoff; Spectrum Labs) against 500 mL of extraction buffer (300 mM NaCl, 50 mM HEPES, pH 7.5) for 12 h at 4°C. The proteins were immediately used in enzymatic assays.

#### Culturing Stool Samples

Approximately 100 mg of frozen stool sample was suspended in 20 mL of pre-reduced PBS and vortexed for homogenization. 500 μL of stool slurry was added to 5 mL of pre-reduced basal cholesterol medium and cultured in an anaerobic chamber at 37°C.

#### Extraction of Cholesterol, Cholestenone, Coprostanone and Coprostanol

Samples (either stool cultures, reaction mixtures with purified enzymes or lysates) were diluted 1:10 in methanol. Insoluble debris was removed by centrifugation (10 min at 5,000 x *g* and 4°C) and the supernatant was injected onto a Kinetex 2.6 μm, C8 100 Å 100 x 3 mm (Phenomenex) column for LC-MS analysis. For the re-analysis of PRISM stool samples (Figure 5C), samples post centrifugation were additionally diluted 1:1 in methanol.

#### Instrumentation and Chromatographic Conditions for Measurement of Sterols

Analysis of the sterols in samples was performed using an ultra-high performance liquid chromatography tandem mass spectrometry (UHPLC-MS/MS) system model Xevo TQ-S (Waters). The mass spectrometer system consists of a triple quadrupole equipped with an atmospheric pressure chemical ionization (APCI) probe. The chromatographic separation was performed on a Kinetex 2.6 μm, C8 100 Å 100 x 3 mm (Phenomenex) column. The LC elution method was as follows: 0–4.5 min (93% B) at a flow rate of 0.5 mL/min at 40°C. Solvent A was water with 0.1% formic acid, and solvent B was acetonitrile with 0.1% formic acid.

To measure cholesterol, cholestenone, coprostanone and coprostanol, the retention times and mass transitions listed below were monitored for each compound: cholesterol (rt 2.70, 369.332 → 147.021), cholestenone (rt 2.60, 385.244 → 108.988), coprostanone (rt 3.00, 369.332 → 147.021 and 387.259 → 369.259), coprostanol (rt 3.20, 371.304 → 95.011).

For the targeted metabolomics method developed for the re-analysis of samples from the PRISM cohort, fecal slurries from 26 stool samples were obtained from the metabolomics platform at the Broad Institute (Franzosa et al., 2019). Samples chosen had a large range of relative abundances for coprostanol as determined by untargeted metabolomics. The only difference in analysis was the LC elution method used: 0–23 min (50% B to 100% B), 23–25 min (100% B), 25-29 min (100% B to 50% B), 29-30  min (50% B), at a flow rate of 0.5 mL/min at 40°C. Solvent A was water with 0.1% formic acid, and solvent B was acetonitrile with 0.1% formic acid. To measure cholesterol, cholestenone and coprostanol, the retention times and mass transitions listed below were monitored for each compound: Cholesterol (rt 17.573, 369.332 → 147.021), cholestenone (rt 17.652, 385.244 → 108.988), coprostanol (rt 18.899, 371.304 → 95.011). Molar ratios of each metabolite were calculated by taking the concentration for each metabolite of interest and dividing by the sum of the concentrations for each of the 3 metabolites measured within a sample. Concentrations of each of the metabolites in a fecal slurry were determined by a standard curve with reference standards.

#### Extraction of DNA and Metagenomic Sequencing of Human Stool Samples

For samples used in Figure 4C, 1 mL of stool culture (described above) at day 3 was centrifuged at 5,000 x g for 10 min. Supernatant was removed and the pellet was frozen until further processing. DNeasy PowerSoil Kit was used to isolate DNA (Qiagen).

For Framingham Heart Study (FHS) samples, stool was collected in 100% ethanol for nucleic acid extraction as previously described (Lloyd-Price et al., 2019). For DNA extraction, a combination of the QIAamp 96 PowerFecal Qiacube HT Kit (Qiagen Cat No./ID: 51531), the Allprep DNA/RNA 96 Kit (Qiagen Cat No./ID: 80311), and IRS solution (Qiagen Cat No./ID: 26000-50-2) kits were used with a custom protocol as previously described (Lavoie et al., 2019). Briefly, approximately 100 mg of stool were transferred into individual wells of the PowerBead plate, with 0.1 mm glass beads (Cat No./ID: 27500-4-EP-BP) prior to bead beating on a TissueLyzer II at 20 Hz for a total of 10 minutes. Samples were transferred into AllPrep 96 DNA plate and processed as per manufacturer's instructions. Purified DNA was stored at -20°C.

For metagenomic library construction, DNA samples were first quantified by Quant-iT PicoGreen dsDNA Assay (Life Technologies) and normalized to a concentration of 50 pg/μL. Illumina sequencing libraries were prepared from 100-250 pg of DNA using the Nextera XT DNA Library Preparation kit (Illumina) according to the manufacturer’s recommended protocol, with reaction volumes scaled accordingly. Prior to sequencing, libraries were pooled by collecting equal volumes (200 nL) of each library from batches of 96 samples. Insert sizes and concentrations for each pooled library were determined using an Agilent Bioanalyzer DNA 1000 kit (Agilent Technologies). Libraries were sequenced on HiSeq 2500 2x101 to yield ~10 million paired end reads per sample. De-multiplexing and BAM and FASTQ file generation were performed using the Picard suite (https://broadinstitute.github.io/picard).

#### Extraction and Sequencing of *E. coprostanoligenes* ATCC51222 and Assembly of High Quality Genome

Cultures of *E. coprostanoligenes* were grown for two days in BCM. Cells were pelleted at 5,000 x g for 10 min and DNeasy PowerSoil Kit was used to isolate DNA (Qiagen). Two different sequencing methods were used to generate sequencing reads for this genome: Nextera XT DNA Library Preparation kit (Illumina) and Oxford Nanopore MinION. For Illumina library construction, see methods above. The second complementary approach used was Oxford Nanopore MinION sequencing using the 1D approach following default Oxford Nanopore protocols for library preparation. Sequencing of *E. coprostanoligenes* on the MinIon was performed with a R9 flow cell resulting in 9527 reads with an N50 length of 2593. Prior to assembly, the Illumina reads were trimmed with Trimmomatic 0.36. Spades 3.9.0 was used to perform a hybrid assembly with the Illumina and Oxford Nanopore MinIon reads using the --nanopore option. The Oxford Nanopore MinIon reads were passed to Spades without correction.

#### Untargeted Metabolomics of Fecal Samples

Cholesterol (rt 7.21, m/z 369.3519), cholestenone (rt 7.00, m/z 385.3465), and coprostanol (rt 7.50, m/z 371.3583) could be identified in published metabolomics datasets (PRISM and HMP2) using peak picking software (Progenesis QI). For more information detailing the generation of the two fecal metabolomics datasets, see Franzosa et al. (2019) and Lloyd-Price et al. (2019).

### Quantification and Statistical Analysis

Raw sequencing data for PRISM (Franzosa et al., 2019), HMP2 (Lloyd-Price et al., 2019), CVON (Kurilshikov et al., 2019), 500FG (Schirmer et al., 2016) and a study by Jie et al. (2017) were downloaded from Sequence Read Archive (SRA): PRJNA400072 (PRISM), PRJNA398089 (HMP2), PRJNA319574 (500FG), from European Genome-Phenome Archive: EGAS00001003508 (CVON), or European Nucleotide Archive (ENA): PRJEB21528 (study by Jie et al).

The quality control for all metagenomic datasets was conducted using Trim Galore! to detect and remove sequencing adapters (minimum overlap of 5 bp) and kneadData v0.7.2 to remove human DNA contamination and trim low-quality sequences (HEADCROP:15 SLIDINGWINDOW:1:20), retaining reads that were at least 50bp.

We employed a two-step approach to analyze metagenomic data: 1) *de-novo* assembly, gene catalogue construction and metagenomic species binning to prioritize functionally and taxonomically interesting enzymes correlated with coprostanol detection in stool metabolomics from PRISM (Franzosa et al., 2019) and HMP2 (Lloyd-Price et al., 2019); and 2) targeted assembly across prioritized samples to create draft genomes for human gut microbes that encode the homologs to the prioritized cholesterol dehydrogenase from *E. coprostanoligenes*.

In step 1, metagenomic reads from all cohorts were assembled individually for each sample into contigs using MegaHIT (Li et al., 2015), followed by an open reading frame prediction with Prodigal (Hyatt et al., 2010) and retaining only full length genes (containing both start and stop codon). A non-redundant gene catalogue was constructed by clustering predicted genes based on sequence similarity at 95% identity and 90% coverage of the shorter sequence using CD-HIT (Fu et al., 2012, Qin et al., 2010). Reads were mapped to the gene catalogue with BWA (Li and Durbin, 2009), filtered to include strong mappings with at least 95% sequence identity over the length of the read, counted (count matrix) and normalized to transcript-per-million (TPM matrix). Count matrix served as an input for binning genes into metagenomic species pan-genomes (core and accessory genes) using MSPminer with default settings (Plaza Oñate et al., 2019). We annotated the gene catalogue at species, genus and phylum levels with NCBI RefSeq (version May 2018) as described previously (Li et al., 2014). To place MSPs that had no match to any species from NCBI RefSeq on a phylogenetic tree we used PhyloPhlAn with default settings (Segata et al., 2013) and used the support values returned by FastTree (Price et al., 2010) to represent the reliability of each split in the phylogenetic tree (similar to bootstrap values). To perform the sensitivity and specificity analysis for coprostanol detection in stool samples, we first clustered the gene catalogue by grouping proteins with >50% AA identity into clusters of homologous proteins (Suzek et al., 2015) and represented their presence / absence in each sample based on the detection of any protein in the cluster (TPM >0). We used clusters of homologous proteins with at least 1% prevalence in PRISM and HMP2, and for each cluster used its detection to classify samples as coprostanol positive (cluster detected) or negative (cluster not detected). By comparing with the actual metabolomics readout of coprostanol presence or absence in each stool sample, we derived measures of sensitivity (true positives / (true positives + false negatives)) and specificity (true negatives / (true negatives + false positives)) for each cluster that represent how well a given cluster correlates with presence and absence of coprostanol. Proteins found in the microbial genomes of interest: *B. dorei* CL03T12C01 (GCF_001640865.1), *B. longum* NCC2705 (GCF_000007525.1) and *Lactobacillus acidophilus* ATCC 53544 (CP022449.1), were mapped to the clusters of homologous proteins with USEARCH ublast (Edgar, 2010) (min. 50% AA identity, 50%). Similarly, USEARCH ublast (Edgar, 2010) was used to map enzymes of interest to the clusters of homologues, but with a more inclusive similarity cutoffs (min. 25% AA identity, 50% coverage).

In step 2, for the prioritized MSPs we selected human gut microbiomes (at least two per MSP) that had the highest cumulative read-per-kilobase (RPK) count across all MSP genes (counted in step 1) for assembly with SPAdes (Bankevich et al., 2012) in ‘--meta’ mode. We also included the 4 cultured stool samples that showed cholesterol dehydrogenase activity. 6 samples were aborted after two assembly trials due to expected very long runtime (≫48 h), and in their case we reverted to the MegaHIT assemblies from step 1. To construct the draft genomes we used genes binned in the respective MSPs (from step 1) to find (min. 95% identity, min. 50% coverage, USEARCH ublast; Edgar, 2010) and extract contigs encoding them. We evaluated the quality of the draft genomes using completeness and contamination measurements based on lineage specific marker genes with CheckM (‘lineage_wf’ workflow) (Parks et al., 2015). As recommended by CheckM framework, draft genomes with >90% completeness and <5% contamination were considered as near complete (high quality draft genomes) (Parks et al., 2015). Additionally, draft genomes with >50% completeness and <10% contamination were defined as medium quality (Bowers et al., 2017). All-vs-all genome-wide calculation of sequence identity for the draft genomes and the genome of *E. coprostanoligenes* was performed with FastANI (Jain et al., 2018).

To test for detection of *E. coprostanoligenes* in the human gut microbiome we searched for its genes in the assembled gene catalogue (min. 95% identity, USEARCH global alignment; Edgar, 2010) or mapped metagenomic stool samples (as in step 1 above) to the assembled gene catalogue that was augmented with the *E. coprostanoligenes* genes (only added genes with less than 95% identity to other genes in the gene catalogue, USEARCH global alignment; Edgar, 2010). In order to link the near quality draft genomes for IsmA-encoding MSPs to the previous studies, we searched their genes for near identical hits among gene sets from metagenomic species (MGS’es) generated in two gene-centric studies (Nielsen et al., 2014, Plaza Oñate et al., 2019) and two genome-binning studies (Pasolli et al., 2019, Almeida et al., 2019) using global alignment (min. 95% nucleotide identity, USEARCH; Edgar, 2010). MGS was matched to an IsmA-encoding MSP if an overlap with at least 50% genes was observed (Figure S5B; Table S4).

The high-quality draft genomes are available for download from NCBI Genomes Bioproject PRJNA559861.

#### Associations with Blood Lipids and Meta-analysis of Four Studies

We studied the relationship between converter status and blood concentration of total cholesterol, LDL-C and HDL-C in three studies with publicly available shotgun metagenomic sequencing datasets: CVON (Kurilshikov et al., 2019), a study by Jie et al. (2017) and one newly sequenced FHS study. Detailed characteristics of studies are provided in Table S6.

Converter status was coded as a dichotomous variable (converter cases and controls (= “non-converter”)). LDL levels were calculated using Friedwald equation (Friedewald et al., 1972). In each study we performed association analysis using a generalized linear model with a given lipid as outcome and encoder status as a predictor. Age (in years), sex, antibiotic usage (yes/no in FHS and 4 categories in CVON (Table S6), and statin usage (yes/no) were fitted as covariates while optimization was performed using *lm* function in R. CVD status (yes/no) and its interaction with encoder status were additionally included in the model as a sensitivity analysis in studies with available data (CVON and FHS) (Table S6). All participants of the JIE et al. study were not taking antibiotics (Jie et al., 2017). For the Jie et al. study, statin usage was not reported for controls and thus only CVD cases were used in our analyses to avoid confounding of associations due to profound effects of statins on lipid concentrations.

Inverse variance-weighted random-effects meta-analysis implemented in meta R package was used to obtain pooled estimates for relationship between converter status and lipid concentrations across all three studies with between-study heterogeneity calculated using I2 statistics (Higgins and Thompson, 2002) (also meta R package).

#### Relationships between Stool Metabolites and Converter Status

Relationships between stool metabolites and converter status were investigated in the PRISM (Franzosa et al., 2019) and HMP2 (Lloyd-Price et al., 2019) studies. In PRISM and HMP2, data for cholesterol, cholestenone and coprostanol was available. We performed log_10_ transformation (with pseudo count of 1e-5 for zero values) of metabolite data followed by calculation of z-scores by subtracting the mean from each individual value and dividing by the standard deviation. For coprostanol we also created dichotomous variables indicating presence or absence of this metabolite in stool samples.

In PRISM, transformed rescaled values of metabolites were used as outcomes in linear regression models using *lm* function (stats package in R), converter status was included as predictor, while age, gender, antibiotic usage (yes/no) and disease status (non-IBD, CD or UC) were used as covariates. For the dichotomous coprostanol variable we utilized the same model specification, but applied a logistic model using glm function (stats package in R).

Given that in the HMP2 study longitudinal metabolite measurements were available, we utilized mixed effects models to study relationships between converter status and stool metabolite concentrations. Transformed metabolite values were fitted as outcomes and converter status was specified as predictor while subjects were included as random effects to account for correlation between repeated measures (lme function from nlme R package). For the dichotomous coprostanol variable we fitted a logistic mixed effects model including subjects as random effects variable (glmer function from lme4 package in R). Age, gender, antibiotic usage (yes/no) and disease status (non-IBD, CD or UC) were included in all models as covariates (fixed effects) in HMP2 study.

#### Comparison with GWAS Meta-analysis for Lipid Traits

We selected two loci that are known drug targets (*HMGCR* and *PCSK9*) and extracted respective effect sizes from the largest GWAS meta-analysis (Willer et al., 2013) to compare with the effects of the studied *ismA* microbial genes. To make GWAS estimates in SD units comparable with reported effect sizes, we have multiplied beta values from GWAS by SD from FHS study. For example, SD of lipid of interest from the FHS cohort (e.g., 0.92 mmol/L for TC) was multiplied by the effect size per allele in SD units (e.g., 0.068 in SD units for *HMGCR*) to give the effect size in mmol/L (e.g., 0.063 mmol/L per allele in FHS study for *HMGCR*).

## Supplemental Information

Document S1. Figures S1–S6

Table S1. Strains Tested for Coprostanol Formation in Monoculture, Related to Figure 2

Table S2. Amino Acid Sequence of Proteins of Interest in This Study, Related to Figure 3

Table S3. Oligonucleotides Used in This Study, Related to STAR Methods

Table S4. Generation of High Quality Draft Genomes for IsmA-Encoding MSPs and Comparison with Previously Published Studies on Metagenomic Species and Results from Commonly Used Microbiome Analysis Software, Related to Figure 4

Table S5. Results from Associations between All Three Fecal Metabolites and Encoder Status for PRISM and HMP2, Related to Figure 5

Table S6. Summary Statistics for the Three Human CVD Cohorts Used in This Study and Results from the Meta-Analysis Investigating Serum Cholesterol Levels, Related to Figure 6

Document S2. Article plus Supplemental Information
