# Supplementary material for: Cholesterol Metabolism by Uncultured Human Gut Bacteria Influences Host Cholesterol Level
Source: Cell Host Microbe. 2020 Aug 12;28(2):245–257.e6. doi: 10.1016/j.chom.2020.05.013 (PMC7435688; doi:10.1016/j.chom.2020.05.013)
Supplement: Document S1. Figures S1–S6 [file mmc1.pdf]

Cell Host & Microbe, Volume *28*

Supplemental Information

Cholesterol Metabolism

by Uncultured Human Gut Bacteria

Influences Host Cholesterol Level

Douglas J. Kenny, Damian R. Plichta, Dmitry Shungin, Nitzan Koppel, A. Brantley Hall, Beverly Fu, Ramachandran S. Vasan, Stanley Y. Shaw, Hera Vlamakis, Emily P. Balskus, and Ramnik J. Xavier

SUPPLEMENTAL INFORMATION

SUPPLEMENTAL FIGURES

Figure S1. Replication of bioinformatic analysis of assembled human gut microbiome gene catalogue in an independent dataset with paired stool metagenomes and metabolomes along with further analysis of the clusters of proteins with homology to proteins encoded by gut microbes previously implicated in coprostanol formation.

Figure S2. Cholesterol metabolism by Eubacterium coprostanoligenes in pure culture and lysates; in vitro activity of purified N-terminal His-tagged ECOP170 expressed in E. coli BL21(DE3); and mechanism and catalytic residues for hydroxysteroid dehydrogenases.

Figure S3. Identification of human-associated cholesterol dehydrogenases with homology to IsmA in the combined assembly of gut metagenomes from six human cohorts.

Figure S4. IsmAs from human-associated gut microbes metabolize cholesterol and coprostanol but not primary bile acids.

Figure S5. IsmA-encoding species identified across 6 human gut microbiome cohorts are related to known Clostridium species and are associated with coprostanol formation in ex vivo stool cultures.

Figure S6. Quantitative targeted metabolomics of cholesterol, cholestenone and coprostanol in a subset of stool samples from the PRISM cohort.

SUPPLEMENTAL FIGURES

Figure S1. Replication of bioinformatic analysis of assembled human gut microbiome gene catalogue in an independent dataset with paired stool metagenomes and metabolomes along with further analysis of the clusters of proteins with homology to proteins encoded by gut microbes previously implicated in coprostanol formation, related to Figure 2. (A-D) Legend as in Fig. 2, apart from value in panel **(A)** where 2.5% of protein clusters are found with greater than 50% specificity and sensitivity. **(E)** Clusters containing proteins with >50% AA identity to a protein found within a specified organism were highlighted on the association figure generated for two independent human cohorts, dataset 1 (PRISM) and dataset 2 (HMP2). **(F)** Scores of sensitivity in relation to presence of coprostanol were very low when calculated for each member of the cluster of homologous proteins that was confirmed to contain cholesterol dehydrogenase enzymes (compare with sensitivity for cluster marked as ECOP170 in Fig. 2D and Fig. S1D).

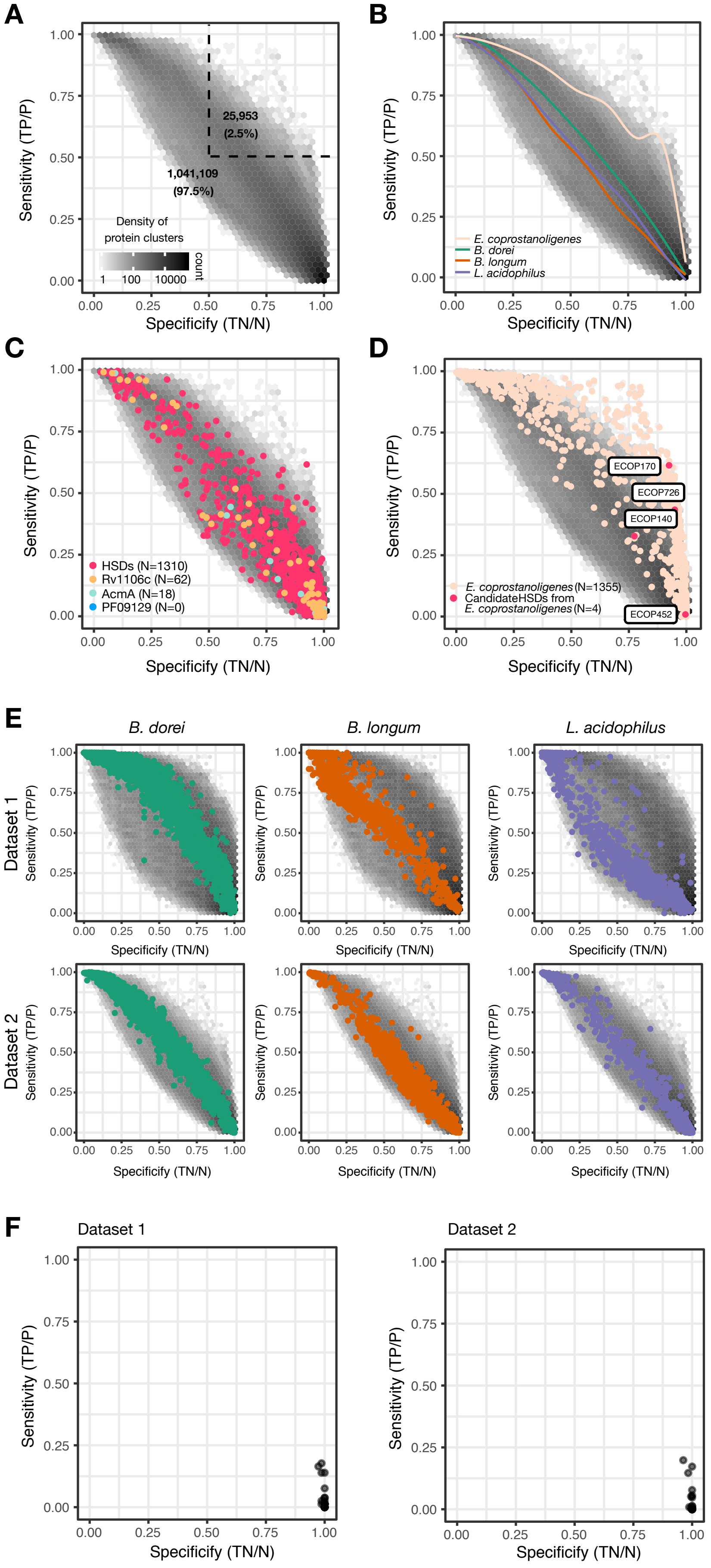
A
B
C
D
E
F
Sensitivity (TP/P)
Specificify (TN/N)
25,953
(2.5%)
1,041,109
(97.5%)
Density of
protein clusters
count
1
100
10000
E. coprostanoligenes
B. dorei
B. longum
L. acidophilus
HSDs (N=1310)
Rv1106c (N=62)
AcmA (N=18)
PF09129 (N=0)
ECOP170
ECOP726
ECOP140
ECOP452
E. coprostanoligenes (N=1355)
CandidateHSDs from
E. coprostanoligenes (N=4)
Dataset 1
Dataset 2

Figure S2. Cholesterol metabolism by *Eubacterium coprostanoligenes* in pure culture and lysates and *in vitro* activity of purified N-terminal His-tagged ECOP170 expressed in *E. coli* BL21(DE3), related to Figure 3. (A) *E. coprostanoligenes* was cultured for 6 days in basal cholesterol medium under anaerobic conditions at 37 °C. At days 2, 4 and 6, samples were taken from the culture and levels of cholesterol and downstream metabolites were quantified using LC-MS. **(B)** Activity assay in *E. coprostanoligenes* clarified lysate after growth for 2 days in media with 2 different buffer conditions. Conversion of cholesterol (100 μM) to cholestenone when a mixture of NAD^+ and $NADP^+$ (100 μM) is added to the clarified lysate occurs in both anaerobic and aerobic assay conditions after overnight incubation in both buffer conditions. **(C)** Activity assay in *E. coprostanoligenes* lysate after growth for 2 days in media without cholesterol and lysis under anaerobic conditions. Conversion of cholesterol (100 μM) to cholestenone occurs under anaerobic conditions in HEPES (50mM, pH = 7.5, 300 mM NaCl) when $NADP^+$ (100 μM) is added to the lysate. Extracted ion chromatograms (EICs) of **2** (rt 2.60, 385.244 → 108.988) are shown for all conditions. **(D)** The expression levels of the four genes encoding putative HSDs in *E. coprostanoligenes* when grown in cholesterol containing medium compared to medium without cholesterol at 48 h by qPCR. *ECOP170* is the HSD with the highest fold change in expression when *E. coprostanoligenes* is grown on cholesterol containing medium compared to medium without cholesterol. Each point represents a biological replicate with the center bar representing the mean and error bars represent s.d. ($n = 3$). P values were determined by one-way ANOVA followed by Tukey's multiple comparisons test. **(E)** N-terminal His_6-tagged protein was purified with Ni-NTA Agarose (Qiagen) **(F)** Activity assay with varying concentrations of ECOP170 in HEPES (pH = 7.5, 50 mM, 300 mM NaCl) with 100 μM cholesterol and either 200 μM of $NADP^+$ (left) or 200 μM of NAD^+ (right) after 10, 20 and 30 min. EICs of **2** (rt 2.60, 385.244 → 108.988) are shown for all conditions. **(G)** Putative mechanism for the oxidation of cholesterol to cholestenone by cholesterol dehydrogenase. **(H)** SDS-PAGE gel showing expression of ECOP170 containing point mutants in E. coli BL21. **(I)** Mutagenesis of ECOP170 shows the predicted active site residues S138, Y151 and K155 are required for conversion of cholesterol to cholestenone.

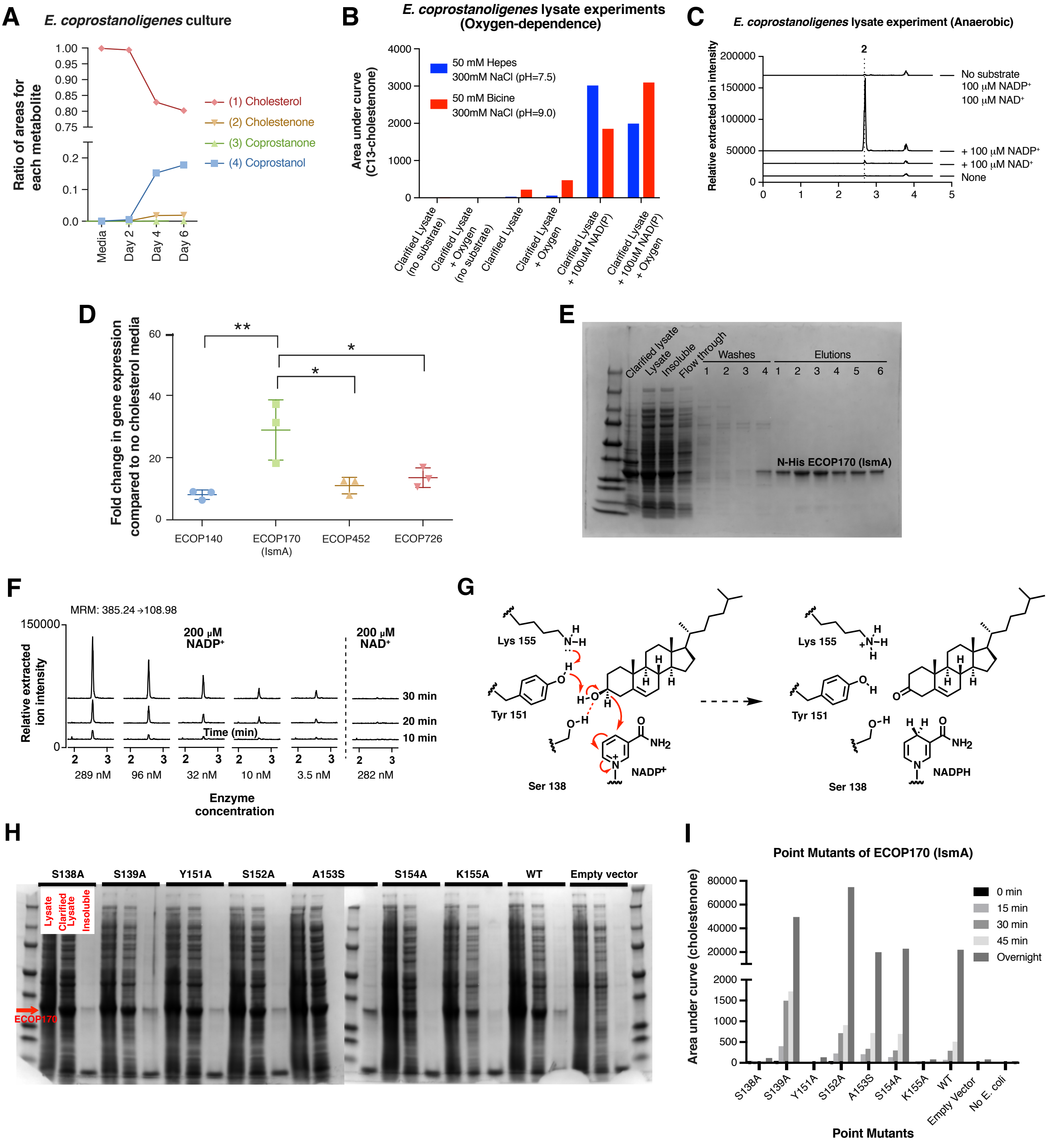
A
E. coprostanoligenes culture
Ratio of areas for each metabolite
(1) Cholesterol
(2) Cholestenone
(3) Coprostanone
(4) Coprostanol
Media
Day 2
Day 4
Day 6
B
E. coprostanoligenes lysate experiments (Oxygen-dependence)
Area under curve (C13-cholestenone)
50 mM Hepes 300mM NaCl (pH=7.5)
50 mM Bicine 300mM NaCl (pH=9.0)
Clarified Lysate (no substrate)
Clarified Lysate + Oxygen (no substrate)
Clarified Lysate
Clarified Lysate + Oxygen
Clarified Lysate + 100uM NAD(P)
Clarified Lysate + 100uM NAD(P) + Oxygen
C
E. coprostanoligenes lysate experiment (Anaerobic)
Relative extracted ion intensity
No substrate 100 μM NADP+ 100 μM NAD+
+ 100 μM NADP+
+ 100 μM NAD+
None
D
Fold change in gene expression compared to no cholesterol media
ECOP140
ECOP170 (IsmA)
ECOP452
ECOP726
E
Clarified lysate
Lysate
Insoluble
Flow through
Washes
Elutions
N-His ECOP170 (IsmA)
F
MRM: 385.24→108.98
Relative extracted ion intensity
200 μM NADP+
200 μM NAD+
30 min
20 min
10 min
Time (min)
289 nM
96 nM
32 nM
10 nM
3.5 nM
282 nM
Enzyme concentration
G
Lys 155
Tyr 151
Ser 138
NADP+
NADPH
H
S138A
S139A
Y151A
S152A
A153S
S154A
K155A
WT
Empty vector
Lysate
Clarified Lysate
Insoluble
ECOP170
I
Point Mutants of ECOP170 (IsmA)
Area under curve (cholestenone)
0 min
15 min
30 min
45 min
Overnight
S138A
S139A
Y151A
S152A
A153S
S154A
K155A
WT
Empty Vector
No E. coli
Point Mutants

Figure S3. Identification of human-associated cholesterol dehydrogenases with homology to IsmA in the combined assembly of gut metagenomes from six human cohorts, related to Figure 3. (A) 1,854 proteins with the highest percent AA identity to ECOP170 were identified. A histogram of the genes with an e-value of less than 1E-50 (33 genes) was made. The percent AA identity bins clearly show a gap in sequences between greater than 60% and less than 45% AA identity with ECOP170. The cutoff for putative cholesterol dehydrogenases was therefore set at 60% AA identity. The percent AA identity bins that contain a characterized cholesterol dehydrogenase protein are identified by the black border and diagonal pattern. **(B)** Percent AA identity matrix between all 25 cholesterol dehydrogenase genes found within the combined microbiome assembly. **(C)** A protein sequence similarity network (SSN) was constructed using the 1,854 proteins from the combined assembly with greater than 20% AA identity to ECOP170. Nodes represent proteins with 100% sequence identity. The SSN is displayed with a percent AA identity threshold of 60%. The 25 cholesterol dehydrogenase proteins are circled and shown in light blue.

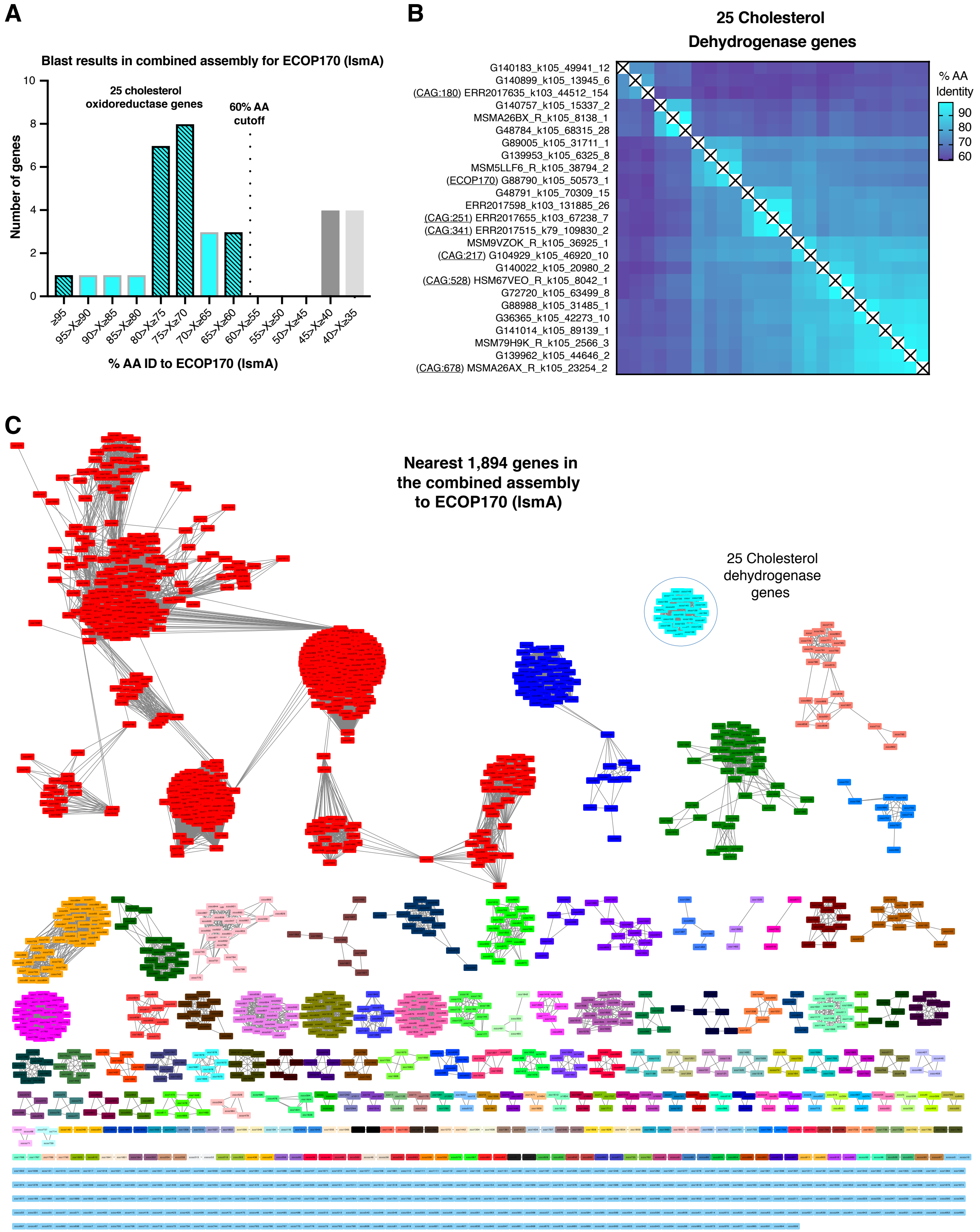

A
Blast results in combined assembly for ECOP170 (IsmA)
25 cholesterol
oxidoreductase genes
60% AA
cutoff
Number of genes
10
8
6
4
2
0
≥95
95>X≥90
90>X≥85
85>X≥80
80>X≥75
75>X≥70
70>X≥65
65>X≥60
60>X≥55
55>X≥50
50>X≥45
45>X≥40
40>X≥35
% AA ID to ECOP170 (IsmA)
B
25 Cholesterol
Dehydrogenase genes
G140183_k105_49941_12
G140899_k105_13945_6
(CAG:180) ERR2017635_k103_44512_154
G140757_k105_15337_2
MSMA26BX_R_k105_8138_1
G48784_k105_68315_28
G89005_k105_31711_1
G139953_k105_6325_8
MSM5LLF6_R_k105_38794_2
(ECOP170) G88790_k105_50573_1
G48791_k105_70309_15
ERR2017598_k103_131885_26
(CAG:251) ERR2017655_k103_67238_7
(CAG:341) ERR2017515_k79_109830_2
MSM9VZOK_R_k105_36925_1
(CAG:217) G104929_k105_46920_10
G140022_k105_20980_2
(CAG:528) HSM67VEO_R_k105_8042_1
G72720_k105_63499_8
G88988_k105_31485_1
G36365_k105_42273_10
G141014_k105_89139_1
MSM79H9K_R_k105_2566_3
G139962_k105_44646_2
(CAG:678) MSMA26AX_R_k105_23254_2
% AA
Identity
90
80
70
60
C
Nearest 1,894 genes in
the combined assembly
to ECOP170 (IsmA)
25 Cholesterol
dehydrogenase
genes

Figure S4. IsmAs from human-associated gut microbes metabolize cholesterol and coprostanol but not primary bile acids, related to Figure 3. (A) SDS-PAGE gels showing expression of all HSDs in *E. coli* BL21. Activity assay with *E. coli* lysates (PBS, pH = 7.4) and either **(B)** 100 µM of cholic acid or **(C)** 100 µM of chenodeoxycholic acid (CDCA) with 100 µM each of NAD^+ and $NADP^+$. TICs are shown for the duration of the method. No reaction was observed with any lysates tested. Activity assay with *E. coli* lysates (PBS, pH = 7.4) expressing one of the IsmA enzymes from human-associated microbes and either **(D)** 100 µM cholesterol or **(E)** 100 µM of coprostanol with 100 µM each of NAD^+ and $NADP^+$. EIC for the products of both reactions are shown.

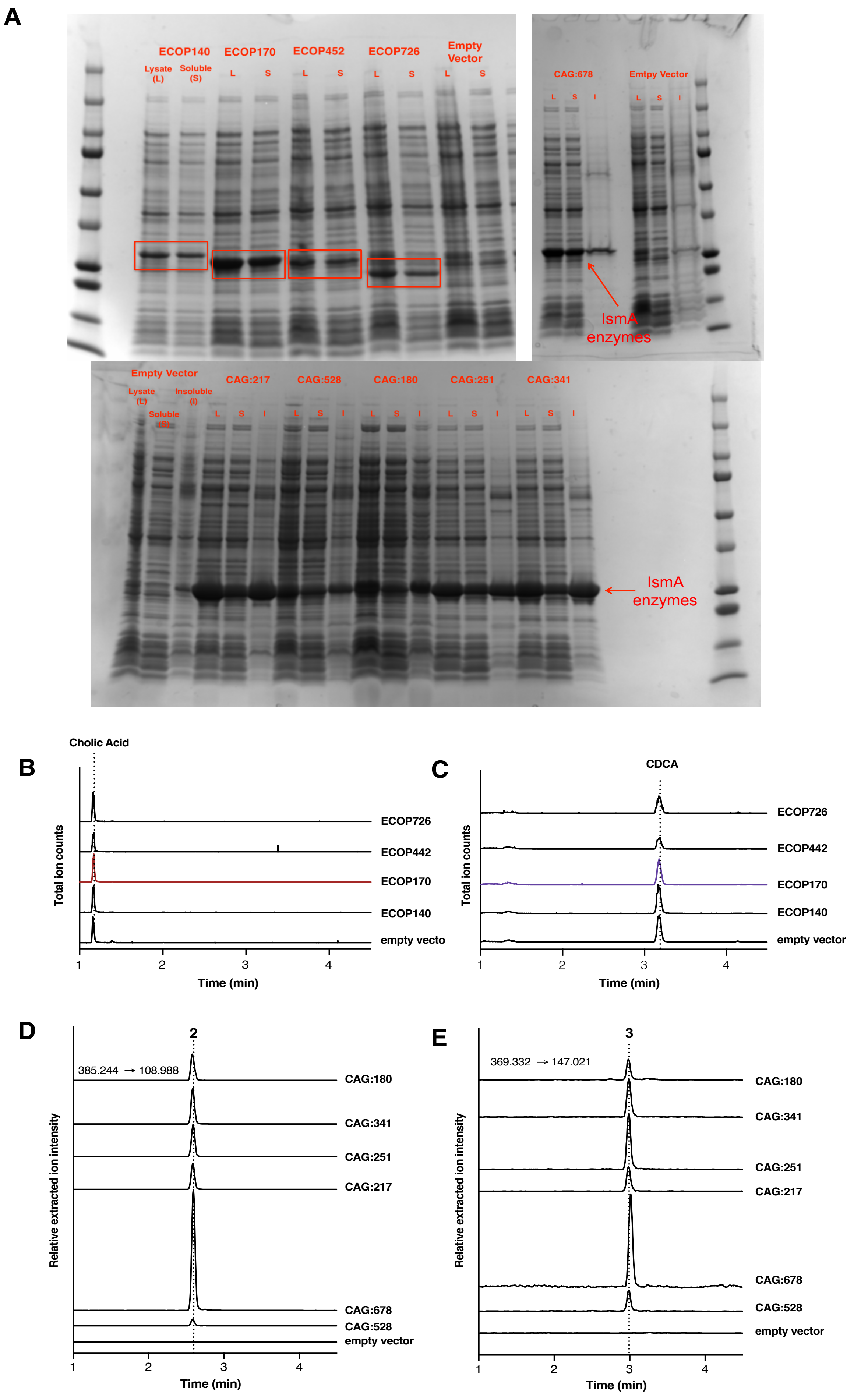
A
ECOP140
Lysate (L)
Soluble (S)
ECOP170
ECOP452
ECOP726
Empty Vector
CAG:678
Emtpy Vector
IsmA enzymes
Empty Vector
Insoluble (I)
CAG:217
CAG:528
CAG:180
CAG:251
CAG:341
B
Cholic Acid
Total ion counts
ECOP726
ECOP442
ECOP170
ECOP140
empty vecto
Time (min)
C
CDCA
empty vector
D
2
385.244 → 108.988
Relative extracted ion intensity
E
3
369.332 → 147.021

Figure S5. IsmA-encoding species identified across 6 human gut microbiome cohorts are related to known *Clostridium* species and are associated with coprostanol formation in *ex vivo* stool cultures, related to Figure 4. (A) Phylogenetic tree as in Fig. 4A with added support values for each split in the tree as calculated by FastTree (Price et al., 2010). **(B)** High quality genomes of IsmA- encoding MSPs were compared with previously published efforts of cataloguing uncultured species (metagenomic species, MGS) in the human microbiome by global alignment at gene level (USEARCH). MSP was assumed "matched" to an MGS if the majority of its genes mapped with min. 95% DNA identity. For this comparison two gene-centric studies (Nielsen et al., 2014); (Plaza Oñate et al., 2018) and two genome-binning studies(Pasolli et al., 2019); (Almeida et al., 2019) were used. **(C)** Relative abundance of the IsmA-encoding MSPs quantified across each dataset used in this study. Boxplots show median and lower/upper quartiles, whiskers show inner fences and dots show outliers. **(D)** Phylogenetic neighbourhood of *Eubacterium coprostanoligenes* including high quality genomes of IsmA-encoding MSPs (orange) and additional 14 IsmA-encoding species from Pasolli et al. and Almeida et al. (blue). Tree was generated using PhyloPhlAn (Segata et al., 2013). **(E)** The average nucleotide identity (ANI) was computed between the MSPs draft genomes and *E. coprostanoligenes* genome. The three MSPs without a high quality draft genome are left blank. ANI scores between two MSPs that are greater than 95% (likely indicating the same species), are marked with a white rectangle.

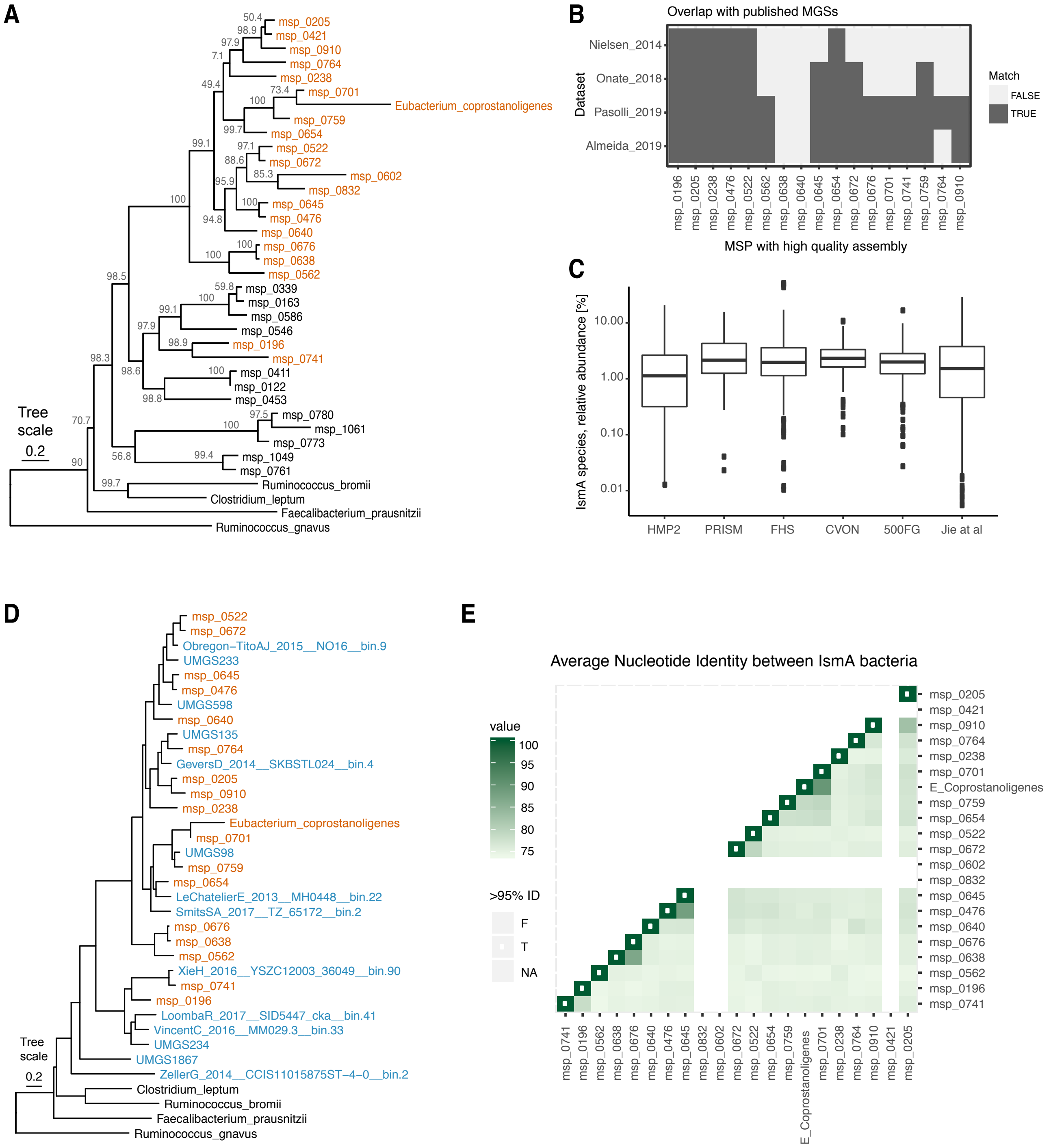
A
Tree scale
0.2
msp_0205
msp_0421
msp_0910
msp_0764
msp_0238
msp_0701
Eubacterium_coprostanoligenes
msp_0759
msp_0654
msp_0522
msp_0672
msp_0602
msp_0832
msp_0645
msp_0476
msp_0640
msp_0676
msp_0638
msp_0562
msp_0339
msp_0163
msp_0586
msp_0546
msp_0196
msp_0741
msp_0411
msp_0122
msp_0453
msp_0780
msp_1061
msp_0773
msp_1049
msp_0761
Ruminococcus_bromii
Clostridium_leptum
Faecalibacterium_prausnitzii
Ruminococcus_gnavus
50.4
98.9
97.9
7.1
49.4
73.4
100
99.7
99.1
97.1
88.6
85.3
95.9
100
94.8
100
100
100
98.5
59.8
100
99.1
97.9
98.9
98.3
98.6
100
98.8
70.7
97.5
100
56.8
99.4
90
99.7
B
Overlap with published MGSs
Dataset
Nielsen_2014
Onate_2018
Pasolli_2019
Almeida_2019
Match
FALSE
TRUE
msp_0196
msp_0205
msp_0238
msp_0476
msp_0522
msp_0562
msp_0638
msp_0640
msp_0645
msp_0654
msp_0672
msp_0676
msp_0701
msp_0741
msp_0759
msp_0764
msp_0910
MSP with high quality assembly
C
IsmA species, relative abundance [%]
10.00
1.00
0.10
0.01
HMP2
PRISM
FHS
CVON
500FG
Jie at al
D
msp_0522
msp_0672
Obregon−TitoAJ_2015__NO16__bin.9
UMGS233
msp_0645
msp_0476
UMGS598
msp_0640
UMGS135
msp_0764
GeversD_2014__SKBSTL024__bin.4
msp_0205
msp_0910
msp_0238
Eubacterium_coprostanoligenes
msp_0701
UMGS98
msp_0759
msp_0654
LeChatelierE_2013__MH0448__bin.22
SmitsSA_2017__TZ_65172__bin.2
msp_0676
msp_0638
msp_0562
XieH_2016__YSZC12003_36049__bin.90
msp_0741
msp_0196
LoombaR_2017__SID5447_cka__bin.41
VincentC_2016__MM029.3__bin.33
UMGS234
UMGS1867
ZellerG_2014__CCIS11015875ST−4−0__bin.2
Clostridium_leptum
Ruminococcus_bromii
Faecalibacterium_prausnitzii
Ruminococcus_gnavus
Tree scale
0.2
E
Average Nucleotide Identity between IsmA bacteria
value
100
95
90
85
80
75
>95% ID
F
T
NA
msp_0205
msp_0421
msp_0910
msp_0764
msp_0238
msp_0701
E_Coprostanoligenes
msp_0759
msp_0654
msp_0522
msp_0672
msp_0602
msp_0832
msp_0645
msp_0476
msp_0640
msp_0676
msp_0638
msp_0562
msp_0196
msp_0741
msp_0741
msp_0196
msp_0562
msp_0638
msp_0676
msp_0640
msp_0476
msp_0645
msp_0832
msp_0602
msp_0672
msp_0522
msp_0654
msp_0759
E_Coprostanoligenes
msp_0701
msp_0238
msp_0764
msp_0910
msp_0421
msp_0205

Figure S6. Quantitative targeted metabolomics of cholesterol, cholestenone and coprostanol in a subset of stool samples from the PRISM cohort, related to Figure 5. (A) Concentrations of cholesterol, cholestenone and coprostanol were determined by comparison to authentic standards in a standard curve (n=26 samples). The values we obtained for each of these metabolites in a given sample were plotted against their respective relative abundances as determined by untargeted metabolomics methods. Cholesterol (rt 17.573, 369.332 → 147.021), cholestenone (rt 17.652, 385.244 → 108.988), coprostanol (rt 18.899, 371.304 → 95.011). Both Pearson and Spearman coefficients (r) are shown for each metabolite. **(B)** The cutoff for calling coprostanol positive samples was determined by using the mean signal plus 3 standard deviations from the 13 coprostanol negative samples as determined by untargeted metabolomics (mean negative samples = 0.0275 mmol of coprostanol/mL of fecal slurry; std. dev of negative samples = 0.0320 mmol of coprostanol/mL of fecal slurry; cutoff for calling samples coprostanol positive = mean of negative sample + 3SD = 0.1236 mmol of coprostanol/mL of fecal slurry). **(C)** Concentrations of cholesterol, cholestenone and coprostanol in each fecal slurry are shown.

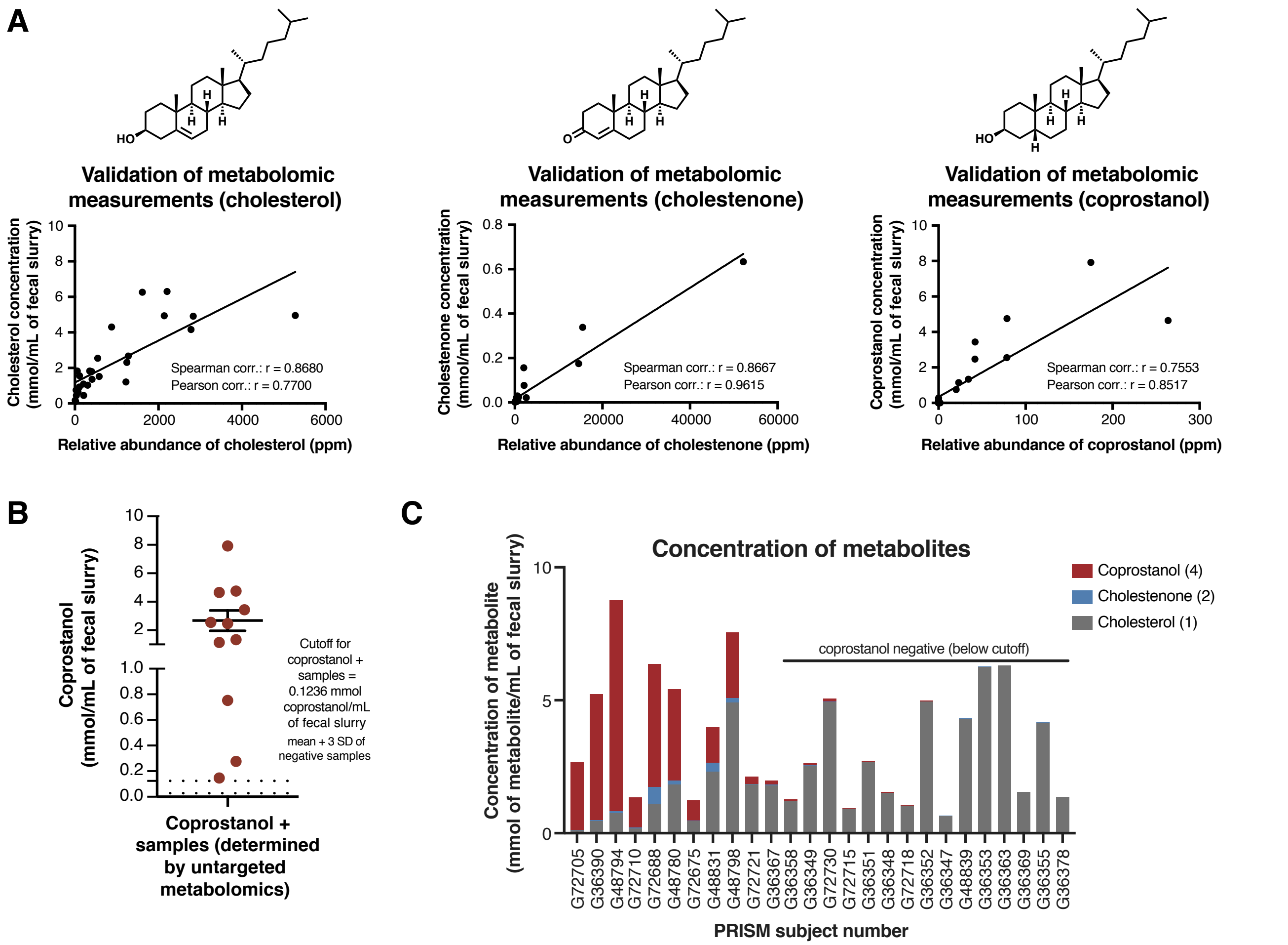

A
HO
H
O
Validation of metabolomic measurements (cholesterol)
Cholesterol concentration (mmol/mL of fecal slurry)
Relative abundance of cholesterol (ppm)
Spearman corr.: r = 0.8680
Pearson corr.: r = 0.7700
Validation of metabolomic measurements (cholestenone)
Cholestenone concentration (mmol/mL of fecal slurry)
Relative abundance of cholestenone (ppm)
Spearman corr.: r = 0.8667
Pearson corr.: r = 0.9615
Validation of metabolomic measurements (coprostanol)
Coprostanol concentration (mmol/mL of fecal slurry)
Relative abundance of coprostanol (ppm)
Spearman corr.: r = 0.7553
Pearson corr.: r = 0.8517
B
Coprostanol (mmol/mL of fecal slurry)
Cutoff for coprostanol + samples = 0.1236 mmol coprostanol/mL of fecal slurry
mean + 3 SD of negative samples
Coprostanol + samples (determined by untargeted metabolomics)
C
Concentration of metabolites
Concentration of metabolite (mmol of metabolite/mL of fecal slurry)
Coprostanol (4)
Cholestenone (2)
Cholesterol (1)
coprostanol negative (below cutoff)
G72705
G36390
G48794
G72710
G72688
G48780
G72675
G48831
G48798
G72721
G36367
G36358
G36349
G72730
G72715
G36351
G36348
G72718
G36352
G36347
G48839
G36353
G36363
G36369
G36355
G36378
PRISM subject number
